# Mobile solutions to Empower reproductive life planning for women living with HIV in Kenya (MWACh EMPOWER): Protocol for a cluster randomized controlled trial

**DOI:** 10.1371/journal.pone.0300642

**Published:** 2024-04-01

**Authors:** Nancy Ngumbau, Jennifer A. Unger, Brenda Wandika, Celestine Atieno, Kristin Beima-Sofie, Julia Dettinger, Emmaculate Nzove, Elizabeth K. Harrington, Agnes K. Karume, Lusi Osborn, Monisha Sharma, Barbra A. Richardson, Aparna Seth, Jenna Udren, Noor Zanial, John Kinuthia, Alison L. Drake

**Affiliations:** 1 Department of Research & Programs, Kenyatta National Hospital, Nairobi, Kenya; 2 Department of Global Health, University of Washington, Seattle, Washington, United States of America; 3 Department of Department of Obstetrics & Gynecology, University of Washington, Seattle, Washington, United States of America; 4 Departments of Biostatistics, Global Health, University of Washington, Seattle, Washington, United States of America; 5 Department of Obstetrics and Gynecology, Women and Infants Hospital, Warren Alpert Medical School of Brown University, Providence, Rhode Island, United States of America; PLOS: Public Library of Science, UNITED KINGDOM

## Abstract

**Background:**

Women living with HIV (WLWH) face unique reproductive health (RH) barriers which increase their risks of unmet need for contraception, contraceptive failure, unintended pregnancy, and pregnancy-related morbidity and mortality and may prevent them from achieving their reproductive goals. Patient-centered counseling interventions that support health care workers (HCWs) in providing high-quality RH counseling, tailored to the needs of WLWH, may improve reproductive health outcomes.

**Methods and design:**

We are conducting a non-blinded cluster randomized controlled trial (cRCT) of a digital health intervention for WLWH (clinicaltrials.gov #NCT05285670). We will enroll 3,300 WLWH seeking care in 10 HIV care and treatment centers in Nairobi and Western Kenya. WLWH at intervention sites receive the Mobile WACh Empower intervention, a tablet-based RH decision-support counseling tool administered at baseline and SMS support during two years of follow-up. WLWH at control sites receive the standard of care FP counseling. The decision-support tool is a logic-based tool for family planning (FP) counseling that uses branching logic to guide RH questions based on participants’ reproductive life plans, tailoring counseling based on the responses. Follow-up SMSs are based in the Information-Motivation-Behavioral (IMB) Skills model of behavioral change and are tailored to participant characteristics and reproductive needs through separate SMS “tracks”. Follow-up visits are scheduled quarterly for 2 years to assess plans for pregnancy, pregnancy prevention, and contraceptive use. The primary outcome, FP discontinuation, will be compared using an intent-to-treat analysis. We will also assess the unmet need for FP, dual method use, viral load suppression at conception and unintended pregnancy.

**Discussion:**

The Mobile WACh Empower intervention is innovative as it combines a patient-centered counseling tool to support initial reproductive life decisions with longitudinal SMS for continued RH support and may help provide RH care within the context of provision of HIV care.

## Introduction

Women and girls in sub-Saharan Africa bear a disproportionately higher burden of HIV than men, with 25% of all the new infections occurring among adolescent girls and young women (AGYW) [1, 2]. HIV affects many dimensions of women’s reproductive health (RH), including reproductive goals and decisions [3]. Women living with HIV (WLWH) experience higher rates of unintended and mistimed pregnancies [4] due to unmet need for contraception, contraceptive failure, and partner-related factors unique to WLWH [5, 6]; resulting in higher risk of maternal morbidity and mortality [7], increased vertical HIV transmission, low birth weight and infant mortality compared to their HIV-negative counterparts [8–10]. While most vertical HIV prevention efforts have focused on maternal HIV treatment and infant prophylaxis (prong 3 of the UNAIDS framework) [11], strategies to reduce unintended pregnancies, unmet need for contraception, and plan for safe conception among WLWH (prong 2) have been under-utilized [12–14].

Unmet need for contraception among WLWH remains high because of many intersecting individual [15, 16], societal [17] and structural barriers [18]. These barriers include insufficient knowledge about family planning (FP), negative family planning beliefs, fear of side effects, partnership dynamics, long wait times at clinics, and stockouts of contraceptive commodities [18, 19]. Additionally, WLWH need to navigate aspects of their reproductive lives related to their HIV infection, such as concerns about drug-drug interactions, achieving viral load (VL) suppression prior to conception, protecting serodiscordant partners from HIV acquisition while trying to become pregnant, and coping with provider and internalized stigma [20–22]. Healthcare worker’s (HCW) own FP beliefs, stigma, as well as lack of training around FP counseling may also contribute to sub-optimal uptake of FP [19–21, 23]. Changes in sexual partnerships and experiences with contraceptive side effects may also contribute to high rates of unmet need for contraception and contraceptive discontinuation rates among WLWH [24, 25].

Innovative interventions providing tailored RH counseling to WLWH that can be embedded within the existing healthcare systems without overburdening HCWs may aid in reducing the unmet need for contraception [26]. Small trials have demonstrated that patient-facing decision-support tools can help women make deliberate and personalized contraceptive choices [27, 28]. Other mobile health (mHealth) technologies, such as SMS-based appointment reminders appear to support reproductive decisions for WLWH as well as provide long-term support for FP use [29]. In two prior trials, a two-way SMS counseling intervention (Mobile WACh; Mobile Solutions for Women’s and Children’s Health) has been shown to improve contraceptive uptake among postpartum women in two randomized trials [30–33]. However, this intervention was originally designed for pregnant and postpartum women seeking care in maternal-child health (MCH) clinics and its primary objective was to support non-FP outcomes. Therefore, it has not been tested to support broader RH goals, delivery within an HIV clinic, or for WLWH specifically. We developed a novel intervention, Mobile WACh Empower, which combines the messaging approach used in the Mobile WACh SMS system with a digital RH decision-support tool to aid in reproductive decision-making and follow-up [27]. Both technologies were adapted to support reproductive life planning among WLWH. We will test the Mobile WACh Empower intervention in a cluster randomized controlled trial (cRCT) to determine the effect of the intervention on contraceptive discontinuation rates, dual method use, and unmet need for FP among women seeking routine HIV care in Kenya. We will also evaluate the acceptability, feasibility, scalability, cost and cost-effectiveness of implementing the Mobile WACh Empower intervention.

## Methods

### Study design and population

The Mobile WACh Empower study is a non-blinded cRCT conducted in 10 HIV clinics in Kenya; 5 rural/peri-urban sites in Western Kenya (Bondo sub-County, Lumumba sub-Country, Rachuonyo District, Siaya District, and Kisumu County Hospitals) and 5 peri-urban/urban sites in Nairobi (Mathare North, Riruta, Kangemi, and Dandora II Health Centres and Kenyatta National Hospital). We will enroll 330 WLWH per facility; eligibility includes: being a WLWH of reproductive age (18–45; 14–17 years if emancipated minor), receiving HIV care at study site and planning to receive care at the enrollment facility for 2 years, having daily access to a mobile phone (own or shared) with Safaricom SIM, and being literate or comfortable with someone reading the study SMS. Pregnant women are ineligible for enrollment.

Prior to study initiation, the study protocol and procedures were approved by the Kenyatta National Hospital Ethics and Research Committee (#P162/03/2022) and the University of Washington Human Subjects Division (STUDY00000438). All participants provide verbal consent prior to eligibility screening and written informed consent prior to trial enrollment.

### Randomization

Facility-level cluster randomization was conducted using a restricted randomization stratified by region to ensure balanced distribution of sites assigned to the intervention and control arms. Five clinics were randomized to receive the Mobile WACh Empower intervention and five to the standard-of-care.

### Intervention

**Fig 1:**
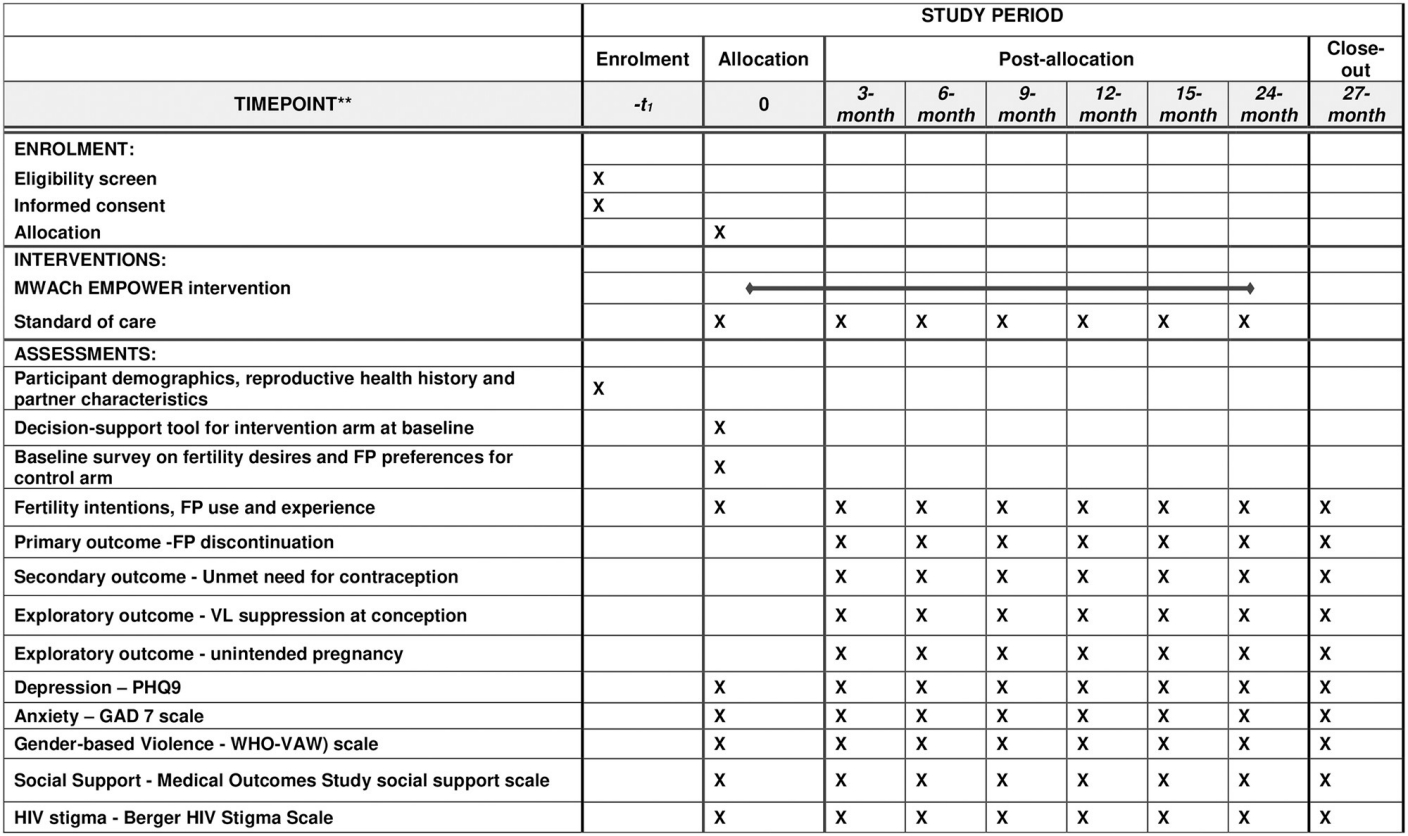
Schedule of enrolment, interventions, and assessments for Mobile WACh Empower study).

The Mobile WACh Empower intervention is comprised of a tablet-based RH decision-support tool and SMS-based communication for education and support. WLWH receiving care at the intervention sites receive the RH decision-support tool at enrollment, and SMS communication for follow-up through 2 years. WLWH at control sites receive standard of care FP counseling and an SMS to welcome them into the study (Fig 1). No follow-up SMS are sent to participants in the control arm.

**Fig 2:**
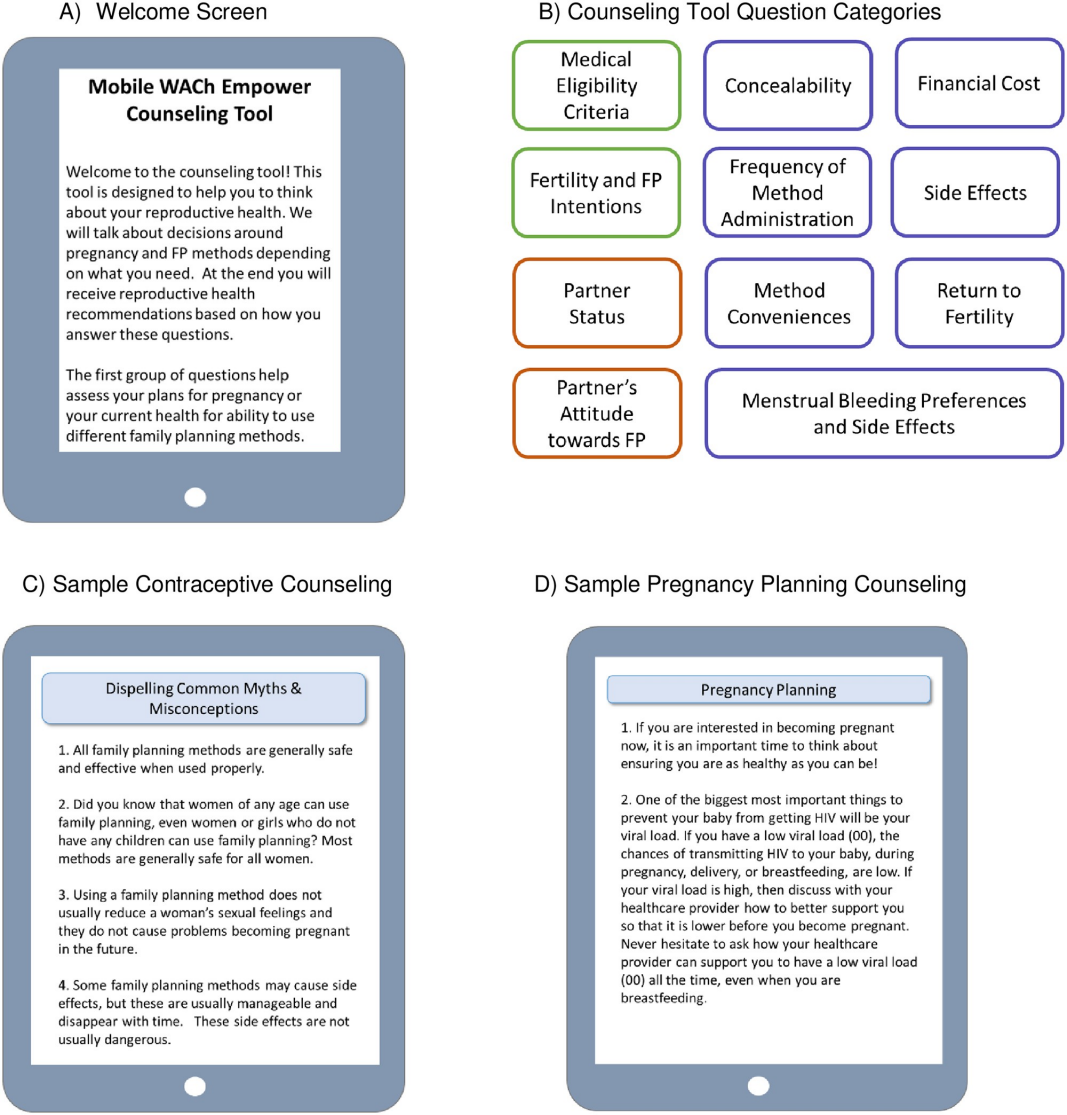
Mobile WACh Empower reproductive life planning counseling tool.

The decision-support tool was adapted from a logic-based tool for FP counseling designed for self-administration, initially pilot-tested with postpartum adolescents and FP providers [34]. This tool was customized specifically for WLWH following stakeholder workshops with WLWH, HCWs, and policymakers and is administered at enrollment as WLWH wait for their routine HIV care visit. The revised decision-support tool includes questions on a variety of topics to guide RH counseling (Fig 2). The first set of questions are related to medical eligibility criteria for contraceptive use, including questions about antiretroviral therapy (ART) use. Next, the decision-support tool asks a series of questions related to fertility intentions, then uses branching logic to guide questions based on participants’ reproductive goals. Subsequent counseling content is tailored based on responses, and importantly, incorporates a spectrum of feelings towards pregnancy, inclusive of pregnancy prevention, ambivalence, and planning. Those who do not desire pregnancy or are ambivalent about pregnancy respond to questions regarding FP preferences and concerns including prior experience and perceptions of each FP method previously used; factors WLWH self-identify as most important in deciding whether to use FP or which FP method to use; partner’s attitudes towards FP; FP method convenience and concealability; tolerability of side effects; method related expenses; frequency of administration; and return to fertility. Finally, educational content addressing negative FP beliefs that are common in Kenya and general education on specific FP methods (oral contraceptive pills, injectables, intrauterine devices, implants, male condoms, sterilization, and/or fertility awareness methods) are provided based on fertility preferences.

**Fig 3:**
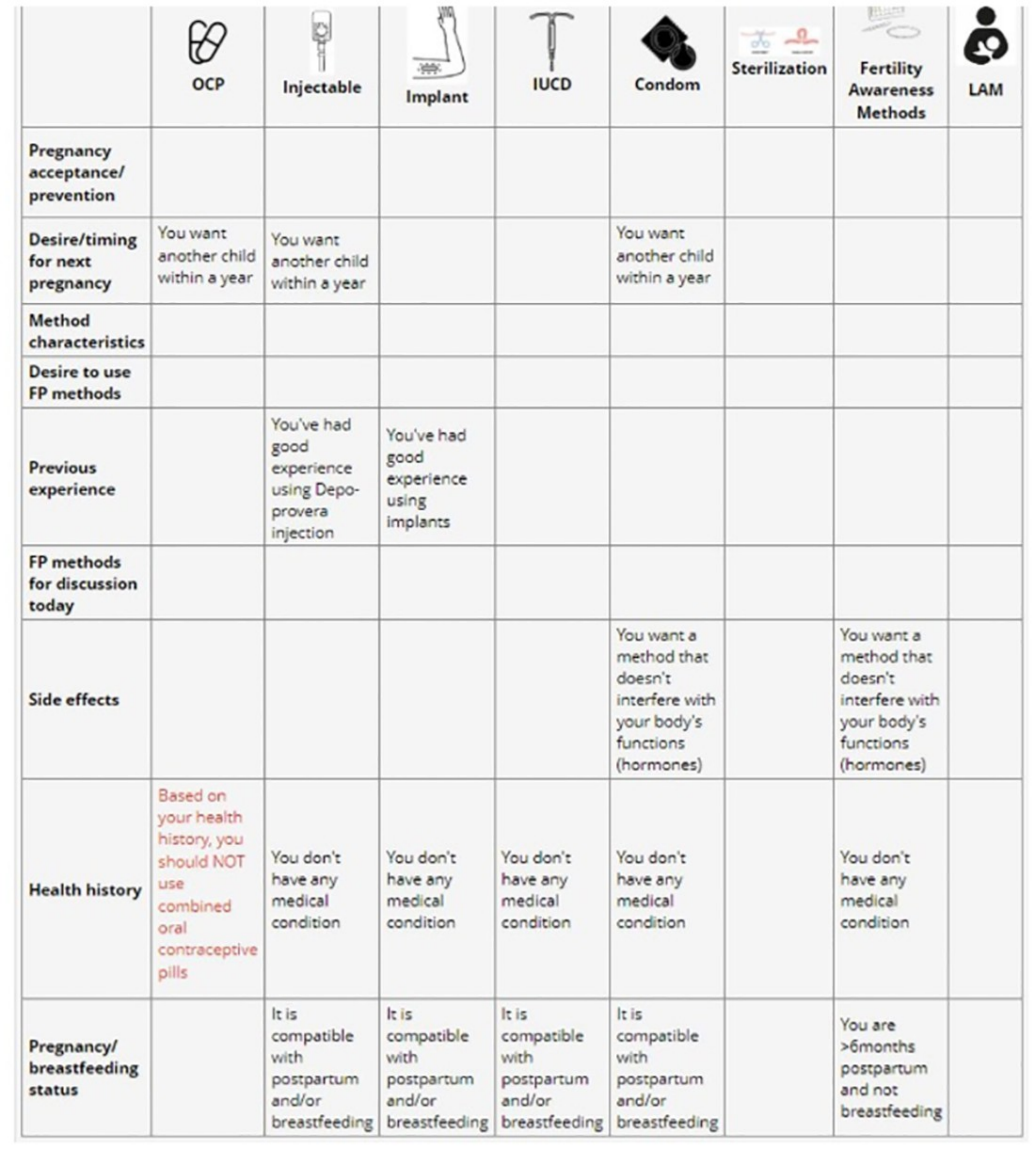
Reproductive life planning counseling tool summary statements, by contraceptive method.

The decision-support tool ends with a summary screen that outlines which method(s) are recommended for them based on their prior responses, as well as a matrix of key categories by FP method (Fig 3 and Table 1). Study staff review the summary screen with participants, who take a printed copy of it to the clinician assigned to provide RH/FP services to WLWH at the facility to continue FP and fertility planning counseling, address any remaining concerns and provide FP services. Women who are planning a pregnancy do not receive a summary screen.

**Table 1:** Example of Mobile WACh Empower reproductive life planning counseling tool summary statements, by contraceptive method.

	Pill	Injection	Implant	IUCD	Condom	Steriliz-ation	FAM	LAM
**Previous experience**	You’ve had good experience using pills	You’ve had good experience using Depo-provera injection	You’ve had good experience using implants	You’ve had good experience using IUCD	You’ve had good experience using condoms			
**FP methods for discussion today**	You would like to discuss pills today	You would like to discuss Depo-provera injection today	You would like to discuss implants today	You would like to discuss IUCD today	You would like to discuss condoms today	You would like to discuss sterilization today	You would like to discuss FAM today	You would like to discuss LAM today
**Side effects**	You are not concerned about using a method that affects your sexual enjoyment or interferes with your body’s functions (hormones)	You are not concerned about using a method that changes your bleeding pattern or causes weight changes or interferes with your body’s functions (hormones)	You are not concerned about using a method that changes your bleeding pattern or interferes with your body’s functions (hormones)	You are not concerned about heavy bleeding due to FP method	You want a method that doesn’t interfere with your body’s functions (hormones)		You want a method that doesn’t interfere with your body’s functions (hormones)	
**Health history**	Based on your health history, you should NOT use combined oral contraceptive pills	You don’t have any medical condition	You don’t have any medical condition	You don’t have any medical condition	You don’t have any medical condition		You don’t have any medical condition	
**Postpartum/ breastfeeding status**	You are >6 weeks postpartum and not breastfeeding	Based on your postpartum and/or breastfeeding status	Based on your postpartum and/or breastfeeding status	Based on your postpartum and/or breastfeeding status	Based on your postpartum and/or breastfeeding status		You are >6 months postpartum and not breastfeeding	You are <6 months postpartum and exclusively breastfeeding, and your period hasn’t returned since your delivery
**Pregnancy acceptance/ prevention**	You are not planning a pregnancy right now and it is not important to prevent one	You are not planning a pregnancy right now and it is not important to prevent one	You are not planning a pregnancy right now	You are not planning a pregnancy right now	You are not planning a pregnancy right now and it is not important to prevent one	You don’t want another child	You are not planning a pregnancy right now and it is not important to prevent one	
**Desire/timing for next pregnancy**	You want another child within a year	You want another child within a year	You want another child after >1 year	You want another child after >1 year	You want another child within a year			
	You are undecided if you want another child				You are undecided if you want another child		You are undecided if you want another child	
**Method characteristics**	You are comfortable taking a pill everyday You are not concerned about someone finding your pills	You are willing to visit a clinic every 3 months for an injection You are not concerned about how quickly you get pregnant after stopping the FP method	You are comfortable using a method that requires seeing a provider or coming into clinic You are not concerned about your partner/family seeing an implant in your arm	You are comfortable using a method that requires seeing a provider or coming into clinic You are not concerned about your partner feeling an IUD (coil) string during sex	Your partner is willing to use condoms			
**Desire to use FP methods**	You don’t want to/ don’t know if you want to use FP but you are not planning a pregnancy				You don’t want to/ don’t know if you want to use FP but you are not planning a pregnancy		You don’t want to/ don’t know if you want to use FP but you are not planning a pregnancy	

**Fig 4:**
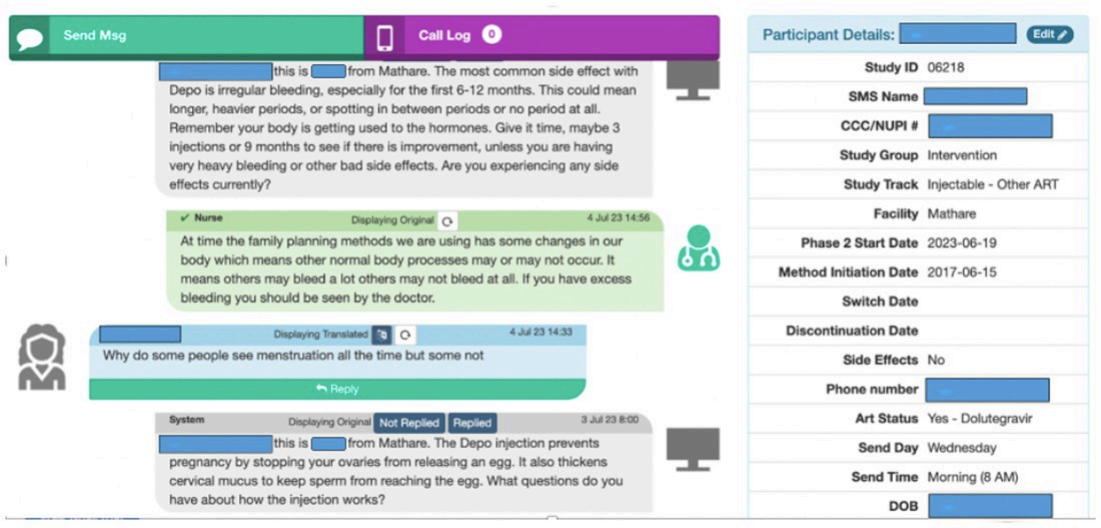
Screenshots of the Mobile WACh human-computer hybrid communication SMS system.

Follow-up SMS communication is administered *via* Mobile WACh, a semi-automated, open-source, cloud-based human-computer hybrid communication SMS system to WLWH in the intervention arm. This system enables both automated SMS to be sent at designated intervals and SMS communication between study nurses and WLWH in the intervention arm (Fig 4) [31, 35]. The system, hosted on a password-protected secure server, can be accessed by study staff on a desktop web browser. It includes a dashboard that displays key participant characteristics, including FP used, FP method initiation/switch/discontinuation date, side effects experienced, age, language, and antiretroviral (ART) regimen used.

**Fig 5:**
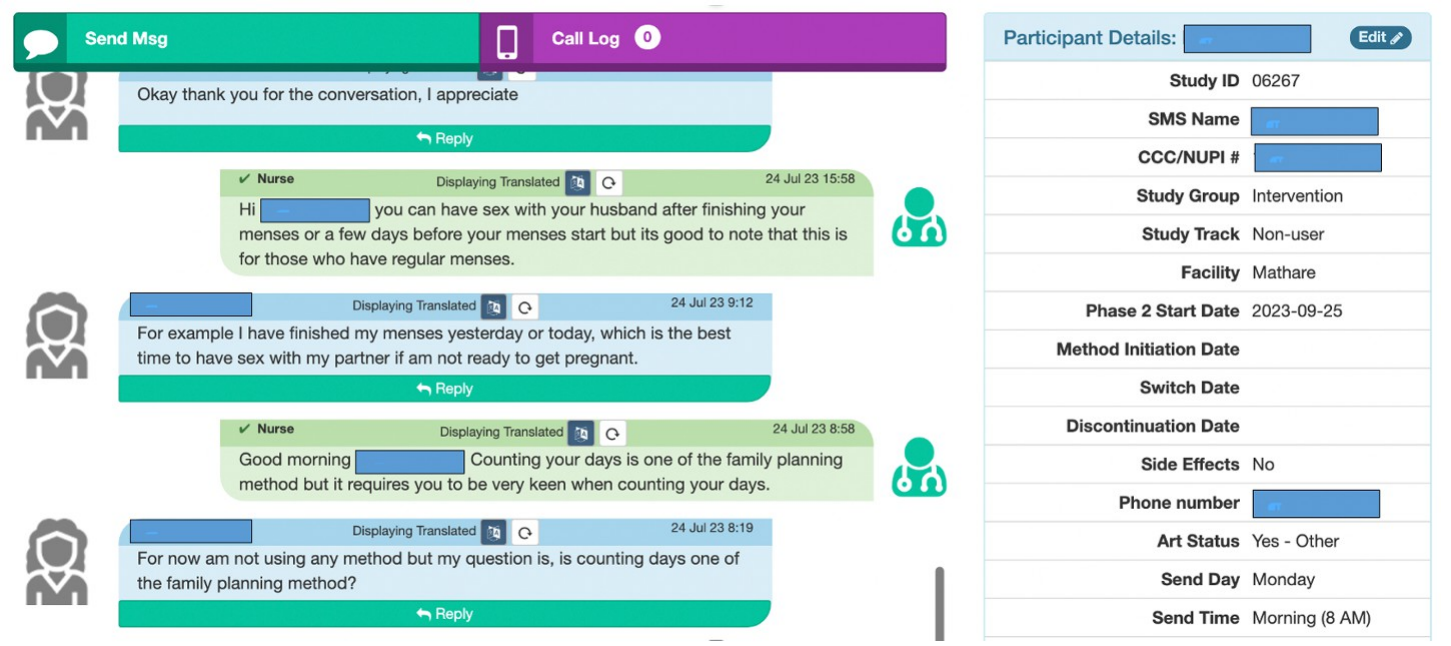
Screenshots of the Mobile WACh system.

These characteristics are captured to assist study staff to appropriately respond to incoming SMS (Fig 5). The system also includes a mechanism to track patient visits and tasks (i.e., SMS needing replies or translations) through a separate dashboard. SMS are preprogrammed into an SMS bank in excel which are uploaded into the SMS system. Automated SMS are then sent to and received by participants free of charge on a reverse-billed short code through a Hypertext Transfer Protocol (HTTP) to SMS gateway maintained by Kenyan premium rate service provider (Safaricom).

**Fig 6:**
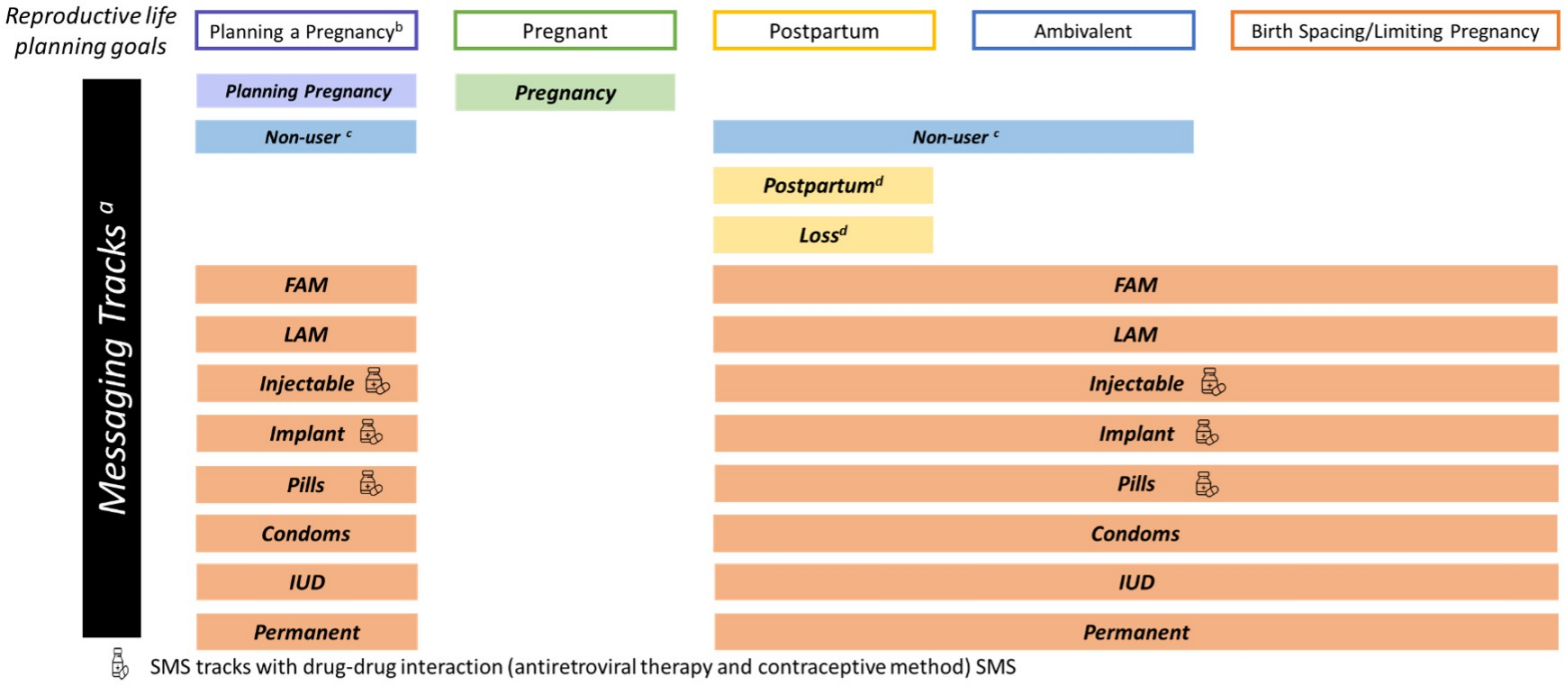
Mobile WACh Empower SMS messaging tracks, by reproductive life planning goals. a) All message tracks include messages based on opt in/opt out HIV messaging and adolescent status (less than 18 years of age).b) Women in reproductive life stage of pregnancy planning include: women who are using FP to prep for safe conception until they are ready to conceive and women who are not using FP and attempting to conceive. c) Women who have discontinued a specific FP method are enrolled in the Discontinuer track that contains the same message curriculum as the Non-user track. d) Women enrolled in the Postpartum track receive tailored messages for 1.5 months and women enrolled in the Loss track receive tailored messages for 1 month. Then women in those tracks are switched to the Discontinuer track or a specific FP method track.

SMS sent to participants in the intervention arm are adapted from the Mobile WACh messaging bank using the Information-Motivation-Behavioral (IMB) Skills model of behavioral change [36, 37] and were revised following feedback from stakeholder workshops with WLWH, HCWs, and policymakers in Kenya conducted prior to study initiation. Selected draft SMS were shared with workshop participants, and feedback was requested on overall content and topics, phrasing and terminology, structure, acceptability, comprehension, and format to guide revisions. The SMS bank has separate RH ‘tracks’ based on fertility desires, FP method, and method discontinuation (Fig 6), and stratified by ART regimen (for selected tracks only). Participants who become pregnant receive specific tracks for pregnancy, postpartum and fetal/infant loss as appropriate following enrollment. Study nurses manually change the participants’ SMS tracks based on their disclosure of changes to their fertility desires, FP method use, discontinuation, or pregnancy status. SMS are further customized into sub-tracks based on whether participants are adolescents, and their HIV-related messaging preferences.

**Fig 7:**
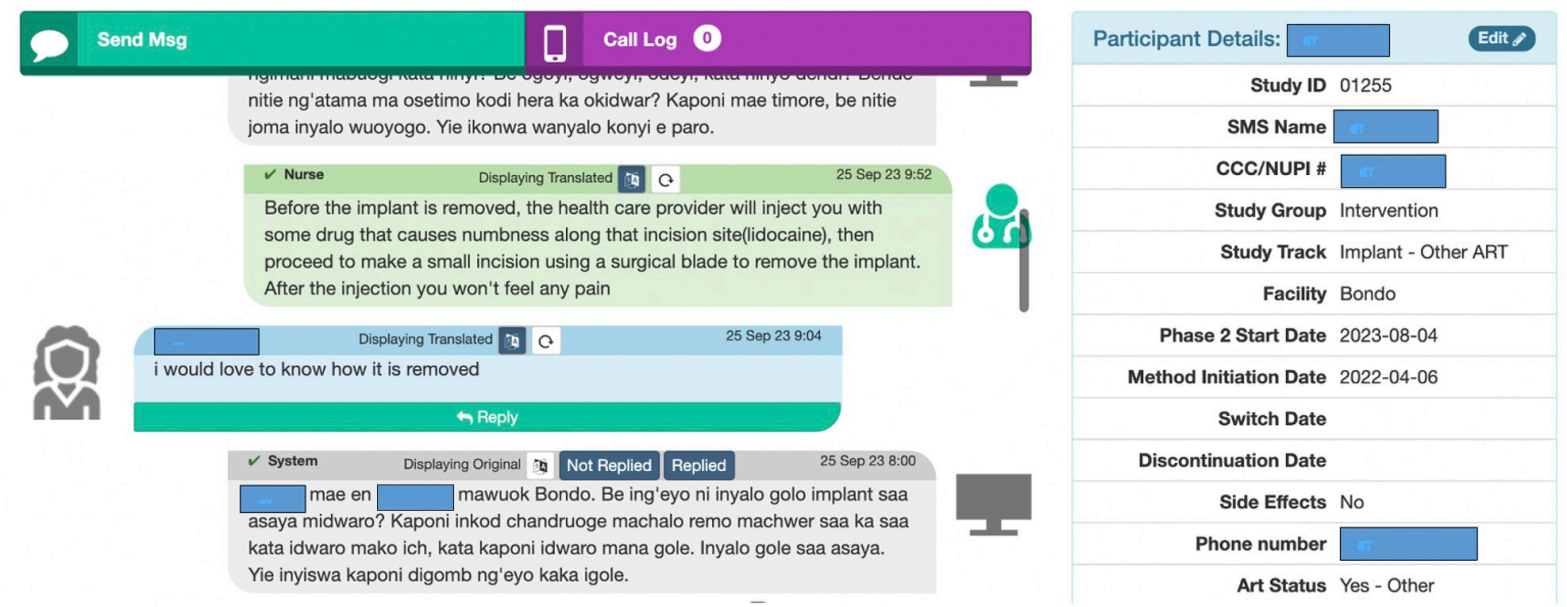
Screenshots of the SMS conversation in Dholuo.

**Fig 8:**
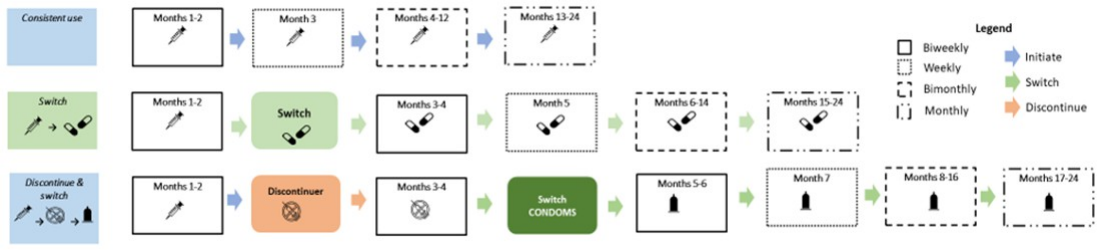
Sample Mobile WACh Empower SMS frequency for contraceptive continuers, switchers, and discontinuers.

Participants are eligible to receive overt HIV SMS if they have disclosed their HIV status to anyone who has access to their phone, or no one has access to their phone; all participants are eligible for covert SMS if they do not want to receive any HIV-related SMS [33]. At enrollment, participants elect their HIV-related SMS content options; options can be changed at any time during the study, according to their eligibility. Participants receive SMS in their preferred language (English, Kiswahili, or Dholou), time of day, and day of the week (Fig 7). SMS messages are sent biweekly through 8 weeks after enrollment, weekly in month 3, bimonthly for months 4–6 then monthly until the end of the trial period. Participants who switch tracks restart this SMS schedule for the new track (Fig 8). Participants can elect to stop receiving all SMS at any time by sending a message with the word, “STOP” to the study short code or requesting the study staff to stop the messages.

### Study procedures

#### Recruitment and screening

WLWH are recruited from the HIV care and treatment and prevention of mother-to-child HIV transmission (PMTCT) clinics at study sites. The ratio of women recruited from the HIV clinic vs PMTCT clinic is site specific and mirrors the distribution of WLWH coming to care at each clinic. After WLWH receive routine HIV/PMTCT care, study staff approach potential participants and briefly tell them about the study. If they are interested, they are screened for eligibility and those who are eligible and interested provide written consent for enrollment.

#### Enrollment, data collection and SMS track assignment

Participants who provide consent are administered electronic surveys to capture demographics, RH and HIV history, and partner characteristics. All participants are entered into the SMS system to receive welcome messages, and scheduled for their next study visit, which aligns with their routine HIV care visit. Since the RH decision-support tool can only be administered after women provide written consent for participation, which occurs after they receive routine HIV care (i.e., after they see a provider), it is not possible to have WLWH receive the decision-support tool at the same visit when consent is obtained. Therefore, enrollment (and receipt of the decision-support tool for participants at intervention sites) for all participants is completed at the next scheduled HIV care visit, scheduled between 1 week and 6 months later, depending on the needs of each WLWH under the differentiated care model in Kenya [38]. At this visit when enrollment is completed, participants at both intervention and control sites are given an Android tablet loaded with a self-administered survey with questions related to fertility desires, FP preferences, and FP concerns. These questions are part of the algorithm used to provide tailored counseling for the intervention arm but are asked in a similar manner (self-administered survey on a tablet) in the control arm so variables can be compared between arms without ascertainment bias. Additional surveys are administered by study staff on ART adherence, depression using the Patient Health Questionnaire-9 [39], anxiety using the Generalized Anxiety Disorder (GAD) 7 scale [40], gender-based violence (GBV) history using the World Health Organization Violence Against Women (WHO-VAW) scale [41], social support system using the Medical Outcomes Study (MOS) social support scale [42], and stigma using the short version of the Berger HIV Stigma Scale (HSS) [43]. Next, participants at intervention sites are assigned an SMS track based on assessment of their RH needs. At the end of the study visit, all participants are scheduled for their next visit which aligns with their routine HIV care visit.

*Follow-up visits*. Follow-up study visits are conducted every 3 months to align with routine HIV care and ART refill visits for 2 years. These visits can be conducted remotely over the phone if the participant is unable to come to the clinic. At each study visit, study staff administer surveys to assess fertility intentions, FP use and experience with FP services, ART adherence, mental health, GBV, social support, and stigma. Participants who report GBV, illnesses, depression or suicidality are referred to available resources within the study facilities and all severe adverse events reviewed by the site-PI and reported to the Kenyan ERC within 48 hours. If participants become pregnant during the study, additional data on pregnancy and delivery/infant outcomes will be captured during follow-up surveys. Data on FP and VL is abstracted from patient records. Additionally, facility-based data is collected monthly from all sites on FP availability and stock-outs; providers’ ability to offer FP methods; clinic volume and staffing levels.

*Acceptability*, *appropriateness*, *feasibility*, *and scalability*. To assess the patient and health system-level acceptability, appropriateness, feasibility, and scalability of implementing the Mobile WACh Empower intervention under real-world conditions, we will conduct focus group discussions (FGDs) with a subset of WLWH and in-depth interviews with HCWs within the intervention sites during the trial period and at exit before the trial ends. Two FGDs will be conducted, stratified by age, at each intervention site (10 WLWH FGDs in total)–one with women <25 years and one with women > = 25 years. Women will be recruited shortly after enrollment (i.e. after administering the counseling tool). During the trial, we will also conduct IDIs with HCWs to understand how they are using the counseling tool to provide reproductive health counseling to WLWH, gather their inputs to improve the counseling tool, and fill a short survey on three implementation outcomes, i.e. acceptability, appropriateness, and feasibility of the counseling tool [44]. From each intervention site, we will interview ~3 HCW who are currently employed at the intervention facility, provide FP services to WLWH, and who’ve interacted with the counseling tool (~15 IDIs in total).

At exit, ten FGDs stratified by RH outcomes (FP continuer, FP switcher, non-pregnant FP discontinuer, pregnant FP discontinuer, pregnancy ambivalence) will be conducted among WLWH and intervention sites will be randomly assigned 2 FGDs from these groups. WLWH will be purposively recruited based on percentile of SMS responses (≤25, >25–75, and >75%) to capture varied SMS user experiences within FGDs. The WLWH FGD guides will be grounded in IMB theory and will evaluate the impact of the intervention on RH decision-making, RH behaviors, including self-efficacy for safe pregnancy, FP use, and FP continuation. For HCWs, 5 FGDs (1 FGD per intervention site) will be conducted. HCW IDI and FGD guides will be grounded in the LMIC-adapted Consolidated Framework for Implementation Research (CFIR) framework to elicit information on individual and health systems influences on acceptability, appropriateness, feasibility and scalability of the intervention. All FGDs will consist of 6–10 people per FGD and will be conducted in English, Kiswahili, or Dhuluo, depending on participant preferences. IDIs with HCWs will be conducted in English. Trained qualitative researchers will administer the IDIs and FGDs and complete a targeted debrief report of the overall impressions. All IDIs and FGDs will be audio recorded, transcribed, and translated into English, if necessary.

*Cost and cost-effectiveness*. Micro-costing and time and motion observation will be used to estimate the incremental costs of implementing the Mobile WACh Empower intervention from a health system perspective. Trial costs will be collected from the facilities using standardized cost menus and the costs divided into start-up, training, clinic space, human resources, supplies and equipment costs. We will also examine study budgets, expense reports, administrative records, and conduct study staff interviews to identify startup and recurrent activities. Research time and other research costs will be excluded from intervention costs. Capital and start-up costs will be annualized assuming a useful life of 5 years and a discount rate of 3%. Prior costs of staff time to operate and respond to Mobile WACh SMS will be extracted from a prior study that used the Mobile WACh SMS system, with costs reviewed and adapted as needed. We will use the contraceptive method mix observed in Mobile WACh Empower, and micro-costing data, to estimate average cost per family planning user, updating a historical model on costs of family planning on PMTCT.

### Sample size

We anticipate 50% of women in the control arm will discontinue FP by 2 years. With 3,300 WLWH (330 women/clinic), assuming α = 0.05, 2-sided testing, a coefficient of variation of 0.2, 10 clinics (5 clinics per arm), and 10% attrition, we have 80% power to detect a 40% reduction in discontinuation for reasons other than planned pregnancy. With 10 clusters, the study has sufficient power to detect a reduction of unmet need for contraception from 25% to 15% and an increase in dual method use from 40% to 65%.

### Outcomes and statistical analysis

The primary study outcome is FP discontinuation, defined as no FP method use for >1 month during the 2 years of follow-up among participants who do not desire a pregnancy. Secondary outcomes include unmet need for FP, defined as desire for pregnancy prevention for 2 years but not using FP, and dual method use, defined as using condoms and another modern FP method (sterilization, oral contraceptive pills, intrauterine devices, injectables, implants, condoms, lactational amenorrhea method, emergency contraceptive pills and diaphragm) [45]. Exploratory outcomes include VL suppression at conception, defined as HIV VL <1000 copies/mL among WLWH who become pregnant or desire pregnancy, and unintended pregnancy, defined as a pregnancy that is mistimed or unwanted, excluding WLWH who have pregnancy ambivalence. Using an intent-to-treat analysis, we will compare the time to FP discontinuation among FP users using Cox-proportional hazards regression, accounting for clustering. Poisson generalized linear models (GLMs) with an offset for time and with robust errors will be used to compare rates of dual method use and unmet need for FP between arms while accounting for clustering. Generalized estimating equations (GEE) and Poisson GLM will be used to compare unintended pregnancy and VL suppression between arms, respectively.

Transcripts from the FGDs will be analyzed using the directed content approach. A codebook based on specific IMB theory (WLWH transcripts), CFIR constructs (provider transcripts), and additional themes identified in debriefing reports will be developed. All transcripts will be coded, and queries used to identify key influences on acceptability, feasibility and future scalability of the Mobile WACh Empower intervention. Thematic network analysis will be used to group identified influences into larger thematic categories and describe individual experiences and health systems considerations, and possible strategies to overcome identified barriers and facilitators.

cRCT outcome data and the micro-costing data will be used to estimate the average cost per FP user. An existing MTCT Markov model used to measure health impact of maternal HIV retesting in Kenya, including upstream elements related to pregnancy risk and unintended pregnancy [46, 47] will be adapted to include estimates of unintended pregnancies including unmet need for FP among WLWH (from Mobile WACh Empower trial), vertical transmission rates in Kenya, current maternal ART and infant ARV regimens, and contraceptive failure rates from LMICs. Model parameters will include time since HIV diagnosis, ART use, ART adherence, HIV viral suppression, breastfeeding status, and infant ARV use. We will utilize the health economics literature to estimate costs of ART provision and other related healthcare expenses. We will project intervention impact on unintended pregnancies and MTCT of HIV. We will calculate incremental cost-effectiveness ratios (ICERs) as the ratio of costs divided by the difference in health outcome (unintended pregnancy) of the Mobile WACh Empower intervention compared to the standard of care over a 20-year time horizon. Consistent with guidelines, we will discount costs and health benefits at 3% annually. We will also conduct sensitivity analyses to identify influential assumptions.

## Discussion

WLWH need strategies that support informed choice and autonomy in RH and consider their diverse RH needs and barriers to care. The Mobile WACh Empower intervention is innovative, as it combines a two-step digital health technology to support initial patient-centered counselling with longitudinal SMS for continued RH support. This intervention may address the dynamic nature of RH needs of WLWH over time with its use of an initial counseling tool with tailored messages based on fertility desires followed by SMS that can be adjusted based on desire for pregnancy and/or method uptake, continuation or discontinuation in real-time. Since it is responsive to the real-life conditions and challenges WLWH face, we hypothesize that the intervention will reduce FP discontinuation among WLWH who do not desire pregnancy and increase dual method use.

Although assessment of fertility intentions and RH needs of WLWH, together with provision of relevant counseling, are important components of comprehensive HIV care [48], integration of these services within the current healthcare systems is challenging [21]. Kenya has made remarkable progress in integrating a range of RH services within HIV care and treatment [49, 50]. However, critical gaps exist, resulting in missed opportunities for WLWH attending HIV care clinics [51]. The Mobile WACh Empower intervention offers a unique model of integrating RH services into existing HIV care spaces by supplementing the current health service paradigm while reducing health-system barriers including lack of time, skills, resources, and training for HCWs [52, 53]. Additionally, the intervention may increase RH service satisfaction and FP self-efficacy since it provides tailored, yet comprehensive, RH counseling and support throughout the continuum of fertility intentions among WLWH [54]. As a result, the quality of RH counseling provided to WLWH may improve, enhancing the experience of WLWH, increasing FP decision-making, and subsequent FP continuation in line with the RH rights of WLWH [55].

While development and use of digital tools for health has increased rapidly over the last few years, <7% of these tools are linked to FP and RH. Among existing FP counseling tools, the majority are designed for English-speaking, Western users; and minimally address individual beliefs, preferences, and social factors influencing FP choice and reproductive goals particularly for WLWH. In addition, counseling tools lack smart-logic to guide counseling and monitor fidelity [34, 56–58], minimizing the ability to assist with initial FP decision-making, reducing overall utility, especially in resource-limited settings. The Mobile WACh Empower intervention recognizes the spectrum of fertility intentions related to reproductive decisions among WLWH and provides comprehensive yet tailored counseling and decision-support based on the values, preferences and individual reproductive plans of WLWH. In addition, the Mobile WACh Empower intervention is context-specific and tailored to the Kenyan setting as it was informed by extensive formative research that was integrated into the content and approach of both the decision-support tool and the SMS.

The Mobile WACh Empower intervention may complement existing facility-based care and provide continuous engagement between WLWH and HCWs without overburdening the healthcare system, particularly in resource-limited settings [32, 33, 59]. The personalized, interactive, and free SMS communication Mobile WACh SMS system can serve as a platform to remotely promote engagement between WLWH and HCWs based on their individual fertility intentions and diverse RH needs, in turn offering continued support between scheduled clinic visits [60]. Thus, the SMS system may provide an opportunity to assess and address the challenges WLWH face while using specific FP methods or planning a pregnancy and allow WLWH to receive solutions in real-time. This may foster contraceptive continuation, reducing unmet need of contraception, reduce the risk of adverse MCH outcomes among WLWH, and support important steps towards ending the HIV epidemic.

### Limitations

The decision-support tool is offered at only one time point, potentially limiting the ability of WLWH to receive this service as their RH needs dynamically change and at key decision points e.g., during FP method switch. Similarly, changing participant track depends on disclosure of current RH conditions to study staff, potentially limiting the real-time applicability of SMS track content. While study participants are not required to be literate, women who are unable to read and write require assistance to administer the RH decision-support tool and read the SMS limiting intervention engagement. Finally, the Mobile WACh Empower SMS is currently limited to subscribers using the largest telecom provider in Kenya, which could limit generalizability, but covers 92% of Kenyans [61].

### Dissemination plans

Study progress and findings will be shared with participating facilities, community advisory board, county leadership and the Kenyan Ministry of Health through policy briefs, written reports and presentations. Study finding will provide guidance on how to integrate FP counseling within existing HIV care and treatment centers and inform future efforts to scale-up the intervention, if effective. We will also share the study protocol and results with other researchers, policymakers, and the scientific community by publishing in peer-reviewed journals and present at local, regional, and scientific conferences.

### Trial status

The cRCT was registered into the clinicaltrials.gov database (#NCT05285670) prior to recruitment. Recruitment and enrollment began on December 1, 2022, and are ongoing. We anticipate completing all follow-up visits by June 2025.

## Supporting information

S1 Checklist SPIRIT 2013 checklist: Recommended items to address in a clinical trial protocol and related documents*. (DOC)

S1 File (DOCX)
