# Supplementary material for: Mobile solutions to Empower reproductive life planning for women living with HIV in Kenya (MWACh EMPOWER): Protocol for a cluster randomized controlled trial
Source: PLoS One. 2024 Apr 1;19(4):e0300642. doi: 10.1371/journal.pone.0300642 (PMC10984530; doi:10.1371/journal.pone.0300642)
Supplement: S1 File — (DOCX) [file pone.0300642.s002.docx]

**TITLE:**

**MOBILE WACh EMPOWER**

**MOBILE SOLUTIONS TO EMPOWER REPRODUCTIVE LIFE PLANNING FOR WOMEN LIVING WITH HIV IN KENYA**

**Contents**

[1. LIST OF ABBREVIATIONS 2](#_Toc97039733)

[2. INVESTIGATORS 3](#_Toc97039734)

[3. COLLABORATING INSTITUTIONS 5](#_Toc97039735)

[4. FUNDING AGENCY 5](#_Toc97039736)

[5. SUMMARY/ABSTRACT 6](#_Toc97039737)

[6. INTRODUCTION/ BACKGROUND 7](#_Toc97039738)

[9. RATIONALE 10](#_Toc97039739)

[10. HYPOTHESIS & STUDY QUESTIONS: 11](#_Toc97039740)

[11. OBJECTIVES 11](#_Toc97039741)

[*11.1 Broad Objectives* 11](#_Toc97039742)

[11.2 Specific Objectives 11](#_Toc97039743)

[12. METHODOLOGY 12](#_Toc97039744)

[AIM 1: Mobile WACh EMPOWER RCT 12](#_Toc97039745)

[12.1 Study Design 12](#_Toc97039746)

[12.2 Study Area Description 12](#_Toc97039747)

[12.3 Study Population 12](#_Toc97039748)

[12.4 Sample Size Determination 13](#_Toc97039749)

[12.7 Study Materials 16](#_Toc97039750)

[12.8 Training Procedures 16](#_Toc97039751)

[12.9 Quality Assurance Procedures 16](#_Toc97039752)

[13. ETHICAL CONSIDERATIONS 16](#_Toc97039753)

[13.1 Consent explanation 16](#_Toc97039754)

[13.2 Institutional Review Board 16](#_Toc97039755)

[13.3 Risks to subjects 16](#_Toc97039756)

[13.4 Protection against risks 17](#_Toc97039757)

[13.5 Potential benefits 18](#_Toc97039758)

[13.6 Compensation 18](#_Toc97039759)

[12.7 Importance of the knowledge to be gained 18](#_Toc97039760)

[14. DATA MANAGEMENT 18](#_Toc97039761)

[15. RESULTS DISSEMINATION 19](#_Toc97039762)

[16. STUDY LIMITATIONS 19](#_Toc97039763)

[16. STUDY TIMELINE 19](#_Toc97039764)

[17. REFERENCES 20](#_Toc97039765)

[APPENDIX I COVID-19 RESPONSE PROCEDURES 28 1](#_Toc97039766)

# 1. LIST OF ABBREVIATIONS

ANC Antenatal Care

ART Anti-retroviral Therapy

CHW Community Health Worker

ERC Ethics Review Committee

FP Family planning

FGD Focused Group Discussion

HIV Human Immunodeficiency Virus

HW Health Worker

IDI In-depth interview

IRB Institutional Review Board

KNH Kenyatta National Hospital

LMIC Low and Middle-Income Countries

M&E Monitoring and evaluation

MCH Maternal and Child Health

MNCH Maternal, newborn and child health

MOH Ministry of Health

MWACh Mobile Solutions for Women’s, Adolescent’s and Child Health

MTCT Mother To Child HIV Transmission

RCT Randomized controlled trial

RH Reproductive Health

SMS Short message service

SOP Standard operating procedure

UoN University of Nairobi

UW University of Washington

WHO World Health Organization

WLWHIV Women Living With HIV

# 2. INVESTIGATORS

Alison Drake, PhD, MPH (PRINCIPAL INVESTIGATOR)

(Responsible for the development, oversight and evaluation of the project)

Assistant Professor

Global WACh Center

Hans Rosling Center Box 351620

3980 15^th^ Ave NE, Seattle, WA 98195

Tel: +1-206-543-5847

Email: adrake2@uw.edu

John Kinuthia, MBChB, MMed, MPH (SITE PRINCIPAL INVESTIGATOR)

(Responsible for development, oversight and science of the project)

Head, Department of Research & Programs, Kenyatta National Hospital

PO Box [20723-00202](tel:20723-00202), Nairobi, Kenya

Tel: +254 0722 799-052

Email: [kinuthia@uw.edu](mailto:kinuthia@uw.edu)

Karume Agnes Karingo, MBChB (CO- INVESTIGATOR)

(Responsible for clinical oversight for development and implementation of intervention)

Research and Programs, Kenyatta National Hospital

PO Box [20723-00202](tel:20723-00202), Nairobi, Kenya

Tel: 0715181283

Email: [karumeagnes@gmail.com](mailto:karumeagnes@gmail.com)

Jennifer A. Unger, MD, MPH (CO-INVESTIGATOR)

(Responsible for assistance in development and science of the project)

Associate Professor, Women and Infants Hospital, Brown University

101 Dudley Street

Providence, RI 02905-299

Tel: +1-401-274-1122

Email: jennifer.unger@brown.edu

Kristin Beima-Sofie, PhD, MPH (CO-INVESTIGATOR)

(Responsible for design of qualitative evaluation and analysis)

Acting Assistant Professor

Department of Global Health

Hans Rosling Center Box 351620

3980 15^th^ Ave NE, Seattle, WA 98195

Tel: +1-206-685-7670

Email: [beimak@uw.edu](mailto:beimak@uw.edu)

Rena Patel, MD, MPH (CO-INVESTIGATOR)

(Responsible for intervention design for HIV content)

Assistant Professor

Department of Medicine, Division of Allergy and Infectious Diseases

Harborview Medical Center

325 9^th^ Ave, Seattle, WA 98104

Email: [rcpatel@uw.edu](mailto:rcpatel@uw.edu)

Monisha Sharma PhD, MPH (CO-INVESTIGATOR)

(Responsible for design of implementation science and costing analysis)

Assistant Professor

Department of Global Health

Hans Rosling Center Box 351620

3980 15^th^ Ave NE, Seattle, WA 98195

Email: [msharma1@uw.edu](mailto:msharma1@uw.edu)

Barbra Richardson PhD (CO-INVESTIGATOR, Scientific mentor)

(Responsible for biostatistic mentorship of the project)

Professor

Departments of Biostatistics, Global Health, Pediatrics, University of Washington

Harborview Medical Center, 325 Ninth Ave., Box 359909, Seattle, WA 98104

Tel: +1-206-543-4278

Email: [barbrar@uw.edu](mailto:barbrar@uw.edu)

Lusi Osborn (DATA MANAGEMENT SPECIALIST)

(Responsible for data collection, data analysis management and scientific plans)

Ahero sub-district Hospital

Ahero, Kenya

Tel: +254 [725 662 840](Tel:0725662840)

Email: [lusiosborn@gmail.com](mailto:lusiosborn@gmail.com)

Celestine Cecilia Atieno

(Kisumu based Research Coordinator; responsible for sites coordination)

Kisumu, Kenya

Tel:0713053766

Email: Celestinececilia31@gmail.com

Brenda Wandika, BSN-RN, MPH (RESEARCH COORDINATOR, Nairobi)

(Responsible for assistance in study coordination, oversight, and implementation)

Department of Medical Research, Kenyatta National Hospital

PO Box [20723-00202](tel:20723-00202), Nairobi, Kenya

Tel: +254725297691

Email: bwandik@uw.edu

Noor Zanial

(Seattle based Research Coordinator; responsible for overall research coordination)

Research Coordinator, Global WACh

Hans Rosling Center Box 351620

3980 15^th^ Ave NE, Seattle, WA 98195

Tel: +1-909-728-1837

Email: [noorz@uw.edu](mailto:noorz@uw.edu)

Aparna Seth, MPP

(Data analyst, Seattle)

(Responsible for assistance in data analysis and management)

Department of Global Health

Hans Rosling Center Box 351620

3980 15^th^ Ave NE, Seattle, WA 98195

Tel: +12062404219

Email: aseth2@uw.edu

Jenna Udren

(Seattle based Research Coordinator; responsible for overall research coordination)

Research Coordinator, Global WACh

Hans Rosling Center Box 351620

3980 15^th^ Ave NE, Seattle, WA 98195

Tel: +1-202-251-1268

Email: jennaiu@uw.edu

# 3. COLLABORATING INSTITUTIONS

- University of Nairobi
- Kenyatta National Hospital
- University of Washington
- Women and Infants Hospital

# 4. FUNDING AGENCY

Funding type: Grant

Name of Funding agency: NIH/ NICHD

**Grant Number**: 1R01HD104551

Principal Investigator on Proposal: Alison Drake

**Title of Proposal:** Mobile WACh Empower: Mobile solutions to empower reproductive life planning for women living with HIV

**Dates:** 06/01/2021-05/31/2026

# 5. SUMMARY/ABSTRACT

Background

Meeting reproductive health (RH) needs for women living with HIV (WLWH) is critical to reduce unintended pregnancies and avert adverse maternal and child health (MCH) outcomes, including prevention of mother-to-child HIV transmission (MTCT). Helping WLWH plan healthy pregnancies is also essential to ensure reproductive justice for this population. In a 2016 national survey in Kenya, we found **integrating RH counseling and service delivery into HIV care** resulted in increased contraceptive use ([1](#_ENREF_1)), consistent with other studies.([2-4](#_ENREF_2)) Yet, **implementation of high quality, women-centered RH services is challenging for HIV providers and health systems who** **lack time, skills, resources, and training.**([5-7](#_ENREF_5)) Tools and “apps” have been developed to facilitate general RH counseling and streamline services, but most lack rigorous evaluation and none have been designed to address unique RH needs and longer term support of WLWH in resource-limited settings.([8](#_ENREF_8), [9](#_ENREF_9))

Significance

While contraceptive initiation is high among WLWH, **30-50% discontinue within 1 year** in sub-Saharan Africa.([7](#_ENREF_7)) Counseling interventions can help mitigate circumstances that lead to discontinuation, a key driver of unmet need for family planning (FP).([10-12](#_ENREF_10)) In addition, **WLWH face** **unique HIV-related issues in reproductive life planning and supporting contraceptive decision-making and management beyond method initiation.** These include choice of antiretroviral treatment (ART) regimen (dolutegravir considerations), drug-drug interactions, side effects, safe conception (viral suppression and protecting serodiscordant partners), and provider and internalized stigma. Interventions to improve counseling and support for fertility planning and contraceptive continuation, tailored for WLWH, will help women make informed decisions to plan, space, or limit pregnancies; better achieve their RH goals; and optimize health outcomes.

Study Aims

Our team has extensively evaluated a **novel 2-way SMS platform (*Mobile WACh*)** **among women receiving MCH services**, that combines automated SMS and 2-way mobile messaging with a health care worker to deliver RH counseling and support in real-time. In 3 trials, Mobile WACh **improved initiation of postpartum contraception**. We propose to adapt Mobile WACh for a **new population** (non-peripartum WLWH), **new environment** (routine HIV care), **and new outcomes for long-term impact** (contraceptive continuation and safe conception). We anticipate this **tailored version of Mobile WACh** will help **provide women with reproductive life planning support to make decisions** and overcome barriers to achieving RH goals. We hypothesize this novel intervention, grounded in behavioral theory, **will reduce contraceptive discontinuation rates and help women safely plan pregnancies**. Counseling and support delivered via SMS directly to women is also expected to reduce the time and number of topics covered during routine counseling, and as a result relieve providers and health care systems of some of the burden to deliver comprehensive RH services and improve quality.

Design

*We will leverage our team’s extensive, interdisciplinary experience on HIV, RH, and mobile health (mHealth) to implement and evaluate the Mobile WACh Empower intervention in a cluster randomized controlled trial (RCT) proceeded by formative workshops with key stakeholders to refine content and approach and post-trial scalability evaluations to ensure potential for real world deployment and scale.*

# 6. INTRODUCTION/ BACKGROUND

Reducing unmet need for family planning (FP), preventing unintended pregnancies, and planning for safe conception among women living with HIV (WLWH) are high priorities for public health. Globally, nearly 18 million women and girls are living with HIV, the majority of whom live in sub-Saharan Africa.([13](#_ENREF_13)) Women in this region also have the highest rate of unmet need for FP, with estimates >35%.([14](#_ENREF_14)) Despite renewed focus on access to FP in resource-limited settings and achieving Sustainable Development Goals (SDGs) related to universal access, **sub-optimal FP use among WLWH continues to lead to unintended pregnancies and elevated risks of mother-to-child HIV transmission (MTCT)**. Helping WLWH plan their reproductive lives, (i.e,. safe conception, prevent unintended pregnancies, and reduce maternal and infant mortality), ([15](#_ENREF_15)) addresses both prongs 2 and 3 of the United Nations comprehensive prevention of MTCT (PMTCT) strategy.([16](#_ENREF_16))

Eliminating unmet need for FP is challenging due to provider, individual, and societal level barriers. Women cite insufficient method knowledge, health concerns, fear of side effects, and partner perceptions as barriers to FP use and continuation; many of which can be addressed with appropriate counseling and continuing support.([17](#_ENREF_17)) FP counseling is a key mediator of FP method selection and use; however, measuring counseling effectiveness is difficult due to variations in content, time, and quality of counseling messages.([18](#_ENREF_18), [19](#_ENREF_19)). **Quality of FP counseling has been identified as a significant programmatic gap in provision of rights-based FP. This includes preconception counseling, women-centered information for FP options, and counseling on method side-effects and general reproductive life planning. (**[**14**](#_ENREF_14)**)**

Contraceptive discontinuation is an important driver of unmet need for FP, and inconsistent use that often precedes discontinuation leads to contraceptive failures.([10](#_ENREF_10)) While some counseling interventions, including a US SMS trial, have reduced discontinuation,([10-12](#_ENREF_10)) counseling interventions that are not tailored based on FP method may be less effective.([20](#_ENREF_20)) In addition, despite one-third of women in LMICs discontinuing FP within a year of initiation, trials to improve FP continuation in LMICs are sparse and none are among WLWH. **Helping WLWH navigate their experience with FP through supportive, tailored SMS counseling has high potential to improve continuation rates for women who want to prevent unintended pregnancies.**

**7. PROBLEM STATEMENT**

Meeting reproductive health (RH) needs for women living with HIV (WLWH) is critical to reduce unintended pregnancies and avert adverse maternal and child health (MCH) outcomes, including prevention of mother-to-child HIV transmission (MTCT). Helping WLWH plan healthy pregnancies is also essential to ensure reproductive justice for this population.

**8. LITERATURE REVIEW**

RH needs extend beyond FP, but decision-making for WLWH is complex due to unique RH needs. WLWH have universal *and* specific RH needs that span beyond pregnancy prevention and PMTCT. **WLWH need holistic reproductive life planning counseling and support to address needs across the RH lifecourse.** Reproductive life plans include a set of personal objectives (based on personal values, goals, and resources) about having or not having children, and the road map to achieve those goals. For WLWH these plans include medications, stigma (both internalized and from community/providers), preconception counseling and options for women who desire or consider pregnancy, as well as FP and PMTCT, as **highlighted by studies below.**

**Box 1. Safer conception strategies**

✓ General health optimization

✓ STI screening and prevention

✓ HIV viral suppression

✓ Optimal ART regimen

✓ DTG counseling

✓ Serodiscordant partner strategies

- *Preconception planning* Considerations for WLWH trying to achieve pregnancy include optimizing HIV management, preventing peripartum complications and MTCT, and safe conception options to prevent transmission for serodiscordant couples([21](#_ENREF_21)) (**Box 1**). For example, preconception counseling on dolutegravir (DTG) use prior to and during pregnancy is warranted, as some women may consider changing ART regimens. In addition, WLWH with serodiscordant partners can plan for safer conception, including ART for viral suppression, partner pre-exposure prophylaxis, and other safer methods.([22](#_ENREF_22))
- *Pregnancy prevention* WLWH must consider concerns about concomitant use of efavirenz and contraceptive implants raised in several studies. Drug-drug interactions can reduce progestin plasma concentrations and increase risks of pregnancy.([23-25](#_ENREF_23)) Providers also need to ensure WLWH have comprehensive information and access to the entire the range of FP methods, including dual method use (male/female condoms plus another modern method of contraception)([26](#_ENREF_26)), in order to meet their reproductive goals; yet, there is evidence that methods are inconsistently offered.([27](#_ENREF_27))

**Meeting WLWH’s RH needs will require listening to the voices of women and incorporating women’s preferences and cultural/contextual factors that are critical drivers of reproductive decision-making.**([28](#_ENREF_28)) Strategies should prioritize addressing explicit and implicit influences on WLWH’s RH decisions, and be convenient and acceptable to women, while simultaneously help **alleviate demands on health systems and HIV providers increasingly asked to provide more services**.

Individual, provider, and health system barriers influence achievement of reproductive life plans. Limited time, inadequate training, and lack of staff and resources are barriers to high-quality women-centered RH counseling and support. Providers may also have misconceptions about FP for WLWH (i.e., ART compatibility and avoidance of intrauterine devices (IUDs)([29](#_ENREF_29)) and emphasize HIV/STI prevention messages and condom use with limited discussion of other methods. Negative provider attitudes and beliefs can create further gaps in service delivery, particularly surrounding safe conception in the context of HIV, which can lead to RH violations for WLWH and couples planning pregnancy.([30](#_ENREF_30), [31](#_ENREF_31)) WLWH and providers have noted a lack of information and tools to optimize care of WLWH during preconception and conception, including accessible guidelines. ([21](#_ENREF_21), [32](#_ENREF_32)) In addition, **women have limited knowledge, internalized stigma, and ambivalence about reproduction and reproductive life plans.(**[**31**](#_ENREF_31)**)**

Integrating RH in HIV care is recommended, but implementation is challenging. It is feasible to co-deliver services under optimal conditions ([33](#_ENREF_33)) and integration can lead to increases in FP use among women in HIV care ([4](#_ENREF_4), [10](#_ENREF_10)). Despite policies supporting integration, reviews suggest implementation is weak and measurement of fidelity is often lacking. HIV providers lack knowledge about FP([34](#_ENREF_34), [35](#_ENREF_35)), and receive little/no training on FP/RH, resulting in inequitable access to RH services in HIV care([36](#_ENREF_36)) and **calls for innovation in service delivery to overcome these health system constraints.**([37](#_ENREF_37)) High-quality provider training is an essential element of successful integration; yet, opportunities for RH training, limited time within routine HIV visits, and staff turn-over create barriers for HIV providers and clinics. **Interventions that minimize additional investments of provider time and knowledge may provide successful, sustainable solutions to optimize RH care for WLWH.**

Integrated service delivery is at the core of addressing the **unfinished agenda of meeting RH needs for women, but the focus of integration at a programmatic level is often too narrow**. FP uptake is a primary metric to demonstrate progress towards meeting RH goals, but it is important to track other indicators to ensure WLWH achieve reproductive life plans over the long term. **Measuring fertility desires and achievement of other RH goals (preconception visits), as well as FP continuation provides an opportunity to track indicators that measure program success more holistically.**

Investment in user-friendly, woman-centered, comprehensive, low cost mobile health (mHealth) tools to reduce barriers to integrating the full range of RH services within HIV care are needed. Innovative approaches to support both women, and the providers who care for them can improve RH for WLWH. RH counseling and decision-support tools improve women’s experiences with FP services and decisions ([38](#_ENREF_38), [39](#_ENREF_39)), and similar **tools should be customized specifically for WLWH**. Tools must incorporate women-centered needs and complexities of WLWH with an efficient and longitudinal approach, meaning women receive tailored counseling in clinic as well as adaptable support when they go home and the state of their world changes.

Many mHealth apps have been developed for English speaking, Western audiences to support FP decision-making.([9](#_ENREF_9), [38](#_ENREF_38), [40](#_ENREF_40)) However, these apps **do not incorporate assessment of the multifaceted environment that affects RH planning and FP use. Most apps require users to select content to access, are inaccessible to most women in resource-limited settings who have basic phones (not smartphones), and are not designed to support WLWH.** Several provider-facing job aids/counseling tools for providers are available to support delivery of FP/RH counseling or offer integrated HIV/RH services in resource-limited settings; ([10](#_ENREF_10), [41](#_ENREF_41)) most are paper-based (i.e., flipcharts or cards) and have not been evaluated. Some tools incorporate decision-algorithms and discussion of both safe conception and FP, but **minimally address individual beliefs, preferences, or personal and social factors influencing FP choice and reproductive life planning for WLWH**. In addition, they often only measure FP uptake or knowledge, and lack smart-logic to guide counseling and intervention fidelity monitoring. Even in settings where tools are available, they are underutilized by providers in low- and middle- income countries (LMICs).([42](#_ENREF_42)) One digital tool supports safe conception for WLWH, but was not designed to address comprehensive reproductive life planning.([22](#_ENREF_22)) **Thus, a novel solution that provides comprehensive, patient-centered RH counseling and support to address barriers important to WLWH in sub-Saharan Africa, is warranted to guide RH decisions and behavior.**

mHealth technologies have potential to improve integrated service delivery and RH outcomes for WLWH, and WHO recommends them to strengthen health systems.([43](#_ENREF_43)) mHealth technology can help reach populations and supplement over-worked providers in resource-limited settings. mHealth interventions in sub-Saharan Africa have successfully improved HIV outcomes, such as ART adherence and reducing HIV VL ([44](#_ENREF_44)), HIV education and HIV testing,([45](#_ENREF_45)) and retention.([46](#_ENREF_46)) The expansion of digital tools for RH also holds promise, **including our Mobile WACh platform of efficient 2-way SMS communication** which improves postpartum FP initiation in MCH settings.([47](#_ENREF_47), [48](#_ENREF_48)) Some RH tools in this space lack evaluations, or fail to detect (or measure) health impact ([49](#_ENREF_49)), and may not be optimized for scale.([20](#_ENREF_20), [50](#_ENREF_50), [51](#_ENREF_51))

**Figure 2.** Screen shot of values and preferences for FP assessed in iMACC counseling tool (*Dev, Reprod Health 2019*)


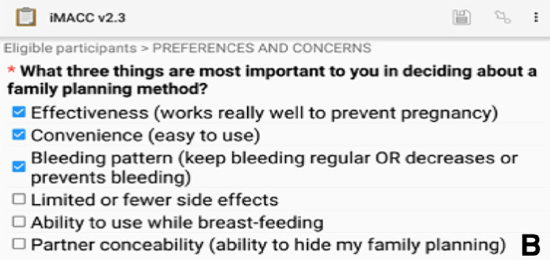


**A well-designed, patient-centered, rigorously evaluated RH counseling intervention will not only address critical RH needs of WLWH but could add efficiency and decrease, rather than add to, provider and health system burden** and ultimately improve RH outcomes. Trials of counseling tools in other fields suggest they are superior to usual care in improving knowledge and setting realistic expectations of benefits and harms, reducing passivity in decision-making, and lowering decisional conflict.([52](#_ENREF_52)) They have also increased satisfaction with FP counseling.([53](#_ENREF_53)) However, current interventions focus on information aggregation and dissemination and fail to fully meet needs of individual patients or achieve sustained behavior change. **WLWH and the providers who care for them need a rigorously designed and evaluated patient-centered mHealth solution that provides holistic and integrated HIV/RH counseling and support for long-term outcomes. We propose to leverage our mHealth experience, and develop and test the Mobile WACh Empower intervention**, to integrate the values, preferences and individual reproductive plans of women by providing tailored counseling and decision-support (FP-related example **Figure 2**) to achieve long-term contraceptive adherence, switching, or discontinuation for pregnancy. Initial counseling in the clinic will focus on these individual domains, and we anticipate the intervention may indirectly result in task-shifting of counseling, alleviating some provider burden to offer comprehensive RH counseling. The intervention will simultaneously streamline and systematize counseling, reducing variation and counseling time on unacceptable or inappropriate FP methods, and improving patient tracking of RH indicators.

Estimating program costs and projecting cost-effectiveness of implementing Mobile WACh Empower can provide important information to decision makers in Kenya when allocating health budgets and deciding which interventions to implement. Mathematical modeling synthesizes costs and intermediate outcomes from trials to project long-term health outcomes of HIV interventions contextualized to local epidemics.([54](#_ENREF_54)) Costing and cost-effectiveness analyses (CEAs) are increasingly used to inform policy decisions and ensure efficient use of limited budgets to maximize population health.

**INNOVATION** Our approach to integrate reproductive life planning and improve RH for WLWH receiving routine HIV care is novel, flexible, scalable, and addresses significant gaps in integrated service delivery. Our behavioral theory-based RH counseling and support intervention, Mobile WACh Empower, will be a highly effective strategy to help WLWH continue with contraception as well as plan, space, and limit pregnancies, and reduce burden on HIV care providers.

Bidirectional support and continued counseling is unique. RH counseling needs are dynamic, changing with experience using FP and evolving fertility desires. Pregnancy ambivalence is also common among WLWH (20%).([55](#_ENREF_55)) **Counseling approaches that not only help WLWH make initial decisions about fertility or using FP but also support desire to start, reconsider, switch or discontinue FP is novel.** Existing provider-facing decision-aids focus on women as unilateral decision-makers, and primarily emphasize FP initiation and method selection; they do not address needs for continual, holistic RH counseling. We propose to develop modules to assess fertility intentions, recognizing there is a spectrum of intentions about reproductive decisions, and provide FP counseling. Counseling messages will be customized to each individual, with built in “memory” linked to patient identifiers, which removes redundancies in messaging at initial and follow-up visits, promotes assessment of challenges using methods, or changes in fertility desires. We will provide the ability for continual counseling, through SMS nurse communication, that recognizes, addresses, and mitigates (where necessary and possible) social and contextual factors that may inhibit WLWH from taking advantage of available RH options.

Approaching RH from the patient-centered view of reproductive life planning for WLWH is novel in LMIC. While many FP programs are focused on outcomes of new FP users and many mHealth tools are designed to support uptake and pregnancy prevention, our counseling and support intervention is designed to meet specific needs of WLWH and the full spectrum of reproductive life plans. This includes supporting women to understand their own reproductive needs, questions or ambivalence and then supporting achieving pregnancy; or choosing contraceptive methods, anticipating and navigating side effects, and committing to (or switching) methods.

SMS to support reproductive life planning for WLWH has high potential for long-term impact on patient outcomes and sustainability. Women’s empowerment is vital to making RH decisions and achieving optimal RH outcomes, and has previously been shown to improve contraceptive use and prevent unintended pregnancies in Africa.([56](#_ENREF_56), [57](#_ENREF_57)) If the intervention helps women gain RH decision-making skills and empowerment, it is highly likely that women can replicate the decision-making process in the future, including being empowered to initiate discussions with providers when they experience RH challenges or want to prepare for pregnancy. These **benefits can extend well beyond the life of the intervention**, and may even extend to other women and girls.([56](#_ENREF_56)) As a result, **the intervention will be durable beyond trial participation, and implementation at scale may require more limited engagement, facilitating the sustainability of this approach**. **In addition, Mobile WACh has been certified a Global Good by Digital Square (PATH) according to the WHO Global Access Policy for Digital Health Tools which endorses its scalability.(**[**58**](#_ENREF_58)**)**

Increased personal understanding of reproductive goals, options, recommendations, and links to health outcomes

Improved insight and increased motivation to use services to achieve RH goals

INFORMATION

BEHAVIORAL SKILLS

Improved skills, practical knowledge, and self-efficacy to effectively implement appropriate reproductive plans

**Outcomes**

↓ Contraceptive discontinuation

↑ Dual contraception

↓ unmet need for FP

MOTIVATION

**Figure 3.** Mobile WACh Empower conceptual framework based on IMB Model for reducing method discontinuation

Rigorous design based in behavioral change theory and long-term outcomes, including the typically under-addressed contraceptive discontinuation. Two major critiques of current mHealth programs are the lack of assessment of intervention effects on critical health outcomes and the lack of intervention design based in behavioral theory.([59](#_ENREF_59)) **This counseling and support intervention is grounded in Information-Motivation-Behavioral (IMB) Skills theory (**[**60**](#_ENREF_60)**) for all outcomes,** improving its potential impact on RH behaviors, generalizability, and likelihood of reproducibility. **Figure 3** represents the conceptual framework for Mobile WACh Empower based on the IMB model of behavioral change which posits that individual health behavior (in this case contraceptive discontinuation without a plan) is predicted by the individuals’ access to information, motivation and behavioral skills to make a plan (continue, switch or plan pregnancy). We hypothesize the intervention supports women through two complementary mechanisms. First, it provides women with *Information and Skills* through counseling, actionable SMS, instrumental support, and interactive triage to prevent, identify, and seek care for contraceptive side effects or other issues. Second, it provides women with *Motivation* though social and health education support ([11](#_ENREF_11), [61](#_ENREF_61)) to continue method use or seek care for alternatives.

# 9. RATIONALE

Meeting reproductive health (RH) needs for women living with HIV (WLWH) is critical to reduce unintended pregnancies and avert adverse maternal and child health (MCH) outcomes, including prevention of mother-to-child HIV transmission (PMTCT). Helping WLWH plan healthy pregnancies is also essential to ensure reproductive justice for this population. In a 2016 national survey in Kenya, we found **integrating RH counseling and service delivery into HIV care** resulted in increased contraceptive use ([1](#_ENREF_1)), consistent with other studies.([2-4](#_ENREF_2)) Yet, **implementation of high quality, women-centered RH services is challenging for HIV providers and health systems who** **lack time, skills, resources, and training.**([5-7](#_ENREF_5)) Tools and “apps” have been developed to facilitate general RH counseling and streamline services, but most lack rigorous evaluation and none have been designed to address unique RH needs and longer term support of WLWH in resource-limited settings.([8](#_ENREF_8), [9](#_ENREF_9))

While contraceptive initiation is high among WLWH, **30-50% discontinue within 1 year** in sub-Saharan Africa.([7](#_ENREF_7)) Counseling interventions can help mitigate circumstances that lead to discontinuation, a key driver of unmet need for family planning (FP).([10-12](#_ENREF_10)) In addition, **WLWH face** **unique HIV-related issues in reproductive life planning and supporting contraceptive decision-making and management beyond method initiation.** These include choice of antiretroviral treatment (ART) regimen (dolutegravir considerations), drug-drug interactions, side effects, safe conception (viral suppression and protecting serodiscordant partners), and provider and internalized stigma. Interventions to improve counseling and support for fertility planning and contraceptive continuation, tailored for WLWH, will help women make informed decisions to plan, space, or limit pregnancies; better achieve their RH goals; and optimize health outcomes.

# 10. HYPOTHESIS & STUDY QUESTIONS:

Our overarching hypothesis is that Mobile WACh Empower, a theoretically grounded counseling tool and two-way SMS intervention that connects WLWH with healthcare workers will improve women’s knowledge, skills and motivation to plan, space or limit pregnancy and achieve better RH goals and health outcomes.

# 11. OBJECTIVES

# *11.1 Broad Objectives*

Our overarching aim is to determine the effect of Mobile WACh Empower on reproductive health outcomes and to understand appropriate pathway to scale for this intervention.

## 11.2 Specific Objectives

We will leverage our team’s extensive, interdisciplinary experience on HIV, RH, and mobile health (mHealth) to implement and evaluate the Mobile WACh Empower intervention in a cluster randomized controlled trial (RCT) in the following aims:

**Aim 1:** To determine the effect of the Mobile WACh Empower intervention (counseling and SMS support) on contraceptive discontinuation rates. Secondary outcomes include dual method use and unmet need for FP.

Approach: We will conduct a cluster RCT of Mobile WACh Empower versus the standard of care (SOC) among WLWH seeking HIV care in 10 facilities in Nairobi and western Kenya followed for 2 years.

*Hypothesis: Women in the intervention arm will have lower contraceptive discontinuation rates and unmet need, and higher dual method use at 2 years compared to the SOC arm.*

**Aim 2:** To evaluate acceptability, appropriateness, feasibility, and scalability of Mobile WACh Empower implementation under real-world conditions.

Approach: We will conduct in-depth interviews and focus group discussions (FGDs) at intervention sites with a subset of WLWH enrolled in Aim 1 and site HIV care providers during the trial and at exit. FGDs with WLWH during the trial and at exit will be grounded in behavioral theory (Information-Motivation-Behavior Skills Theory) to determine patient-level intervention acceptability and feasibility. During the trial, IDIs will be conducted with providers as well as FGDs at exit. These will be grounded in implementation science (Consolidated Framework for Implementation Research) to understand health system-level acceptability, appropriateness, feasibility and plan for scalability.

*Hypothesis: The intervention will support women’s fertility and contraceptive choices, improve RH self-efficacy, meet needs for RH counseling and support, and streamline provider workflow and counseling. Readiness and implementation climate needed for scaling Mobile WACh Empower will also be assessed.*

**Aim 3:** To estimate the cost and cost-effectiveness of implementing Mobile WACh Empower in HIV clinics in Kenya on unintended pregnancies and MTCT. Approach: We will conduct micro-costing and time-motion observations to estimate intervention costs from a health system perspective. We will combine costs and trial outcome data from Aim 1 in a mathematical model to project the health and economic impact of implementing Mobile WACh Empower in Kenya, including costs per unintended pregnancy and MTCT averted.

*Hypothesis:* *Mobile WACh Empower will be a cost-effective approach for preventing unintended pregnancies.*

# 12. METHODOLOGY

Each AIM will have a separate Methodology section

## AIM 1: Mobile WACh EMPOWER RCT

## 12.1 Study Design

The study is a non-blinded cluster randomized controlled trial.

We will conduct a cluster RCT to evaluate a combined counseling intervention (tablet decision-support tool plus SMS for follow-up support) to (counseling and SMS support) on contraceptive discontinuation rates. Cluster RCTs are well-suited to interventions that occur at the clinic, rather than individual level. The randomization at a clinic level allows for the traditional benefits of randomization. We will include a total of 10 clinics in the RCT, 5 receiving standard of care and 5 receiving the intervention and compare contraceptive discontinuation as a primary outcome, and dual method use and unmet need for family planning as secondary outcomes, between the 2 arms.

Randomization procedures:

For the RCT, 10 clinics will be randomized to receive the Mobile WACh Empower intervention or no intervention (SOC). Depending on the heterogeneity of baseline facility factors, restricted randomization may be used to ensure an equal distribution of potentially confounding factors. Dr. Richardson (Biostatistician) will generate the randomization assignment for each clinic.

Masking procedures: Because this is a clinic-level intervention, there will be no masking procedures.

Detailed Description of Procedures:

5 clinics will be randomized to receive the Mobile WACh Empower intervention and 5 to receive no intervention. Depending on the heterogeneity of baseline facility factors, restricted randomization may be used to ensure an equal distribution of potentially confounding factors such as urban vs. rural site or clinic size. Dr. Richardson (Biostatistician) will generate the randomization assignment.

## 12.2 Study Area Description

**Study sites**: The proposed study will be conducted at 10 HIV clinics in western Kenya and Nairobi county. We will select facilities from Bondo sub-County, Lumumba sub-Country, Rachuonyo District, Siaya District, Kisumu District General Hospitals in Western Kenya and Mathare North Health Center, Riruta Health Center, KNH, Kangemi Health Centre, Kibera South Health Center, Dandora 2 Health Centre, Kayole 2 Health Centre and Kariobangi North Health Centre. These facilities represent a mix of rural and urban facilities, all with sufficient patient volume to assure feasibility of recruitment.

## 12.3 Study Population

**Box 2. Eligibility Criteria for WLWH**

- HIV-infected
- Receiving HIV care at study site
- Reproductive age (18-45; 14-17 if emancipated minors)
- Daily access to mobile phone (own phone or shared) with Safaricom SIM
- Plan to receive HIV care at enrollment facility for 2 years
- Speak English, Kiswahili, or Luo
- Literate or comfortable with someone reading study SMS

We will recruit WLWH and HIV providers receiving or providing HIV care, respectively, from 10 HIV clinics. These facilities will include Bondo sub-County, Lumumba sub-Country, Rachuonyo District, Siaya District, and Kisumu District General Hospitals in western Kenya and Mathare North Health Center, Riruta Health Center, KNH, Kangemi Health Centre, Kibera South Health centre, Dandora 2 Health Centre, Kayole 2 Health Centre, Kariobangi North Health Centre in Nairobi. Eligibility criteria are shown in **Box 2**. Adolescents age 14-17 with prior pregnancies are emancipated minors by Kenyan law. Literacy in study regions is high (>90%), but if women have a partner or family member whom they would feel comfortable having read them SMS no exclusions based on literacy will be made to enhance generalizability. This approach has been successful in prior Mobile WACh studies, including WLWH. Currently pregnant women will be excluded; breastfeeding women will be eligible.

## 12.4 Sample Size Determination

| **Table 3. Number of clusters needed assuming n=300 women per cluster (accounting for up to 10% attrition) 80% power, α=0.05, two-sided testing, coefficient of variation=0.2** | | | | | |
| --- | --- | --- | --- | --- | --- |
| *FP discontinuation* ***Arm 1*** | | | | | |
| **Arm 2** | **20%** | **25%** | **30%** | **35%** | **40%** |
| **45%** | 6 | 8 | 12 | 26 | 103 |
| **50%** | 6 | 6 | 10 | 14 | 32 |
| **55%** | 4 | 6 | 8 | 10 | 18 |
| **60%** | 4 | 6 | 6 | 8 | 12 |

Based on literature ([7](#_ENREF_7)) and recent data among women receiving HIV care in Kenya ([63](#_ENREF_63)) we anticipate 50% of women in the control arm will discontinue FP by 2 years. Assuming α=0.05, 2-sided testing, and a coefficient of variation of 0.2, 10 clinics (5 per arm), and 300 women/clinic we have 80% power to detect a 40% reduction in discontinuation for reasons other than planned pregnancy (30% discontinuation rate) (**Table 3**). Prior data suggest the coefficient of variation falls as cluster size increases, is often ≤0.25, but rarely >0.5; therefore, a coefficient of variation of 0.2 is expected to be adequate. We will enroll 347 women to allow for 10% attrition after enrollment plus additional attrition between consenting and the return visit for use of the counseling tool and completion of enrollment, for a total sample size for analysis of 3300 women. Power was conservatively calculated factoring in potential attrition. With 10 clusters we will also have sufficient power to detect a difference in unmet need for FP (reduction from 25% to 15%) and an increase in dual method use from 40% to 65%.

**12.5 Statistical analysis** We will compare facility and individual characteristics (accounting for clustering) to determine adequacy of randomization. In the primary intent-to-treat analysis, we will compare time to FP discontinuation (no method use for >1 month, 2 years of follow-up) using Cox-proportional hazards regression, accounting for clustering.([87](#_ENREF_87)) Women not initially on FP at baseline will enter the analysis once initiating FP. We will use Poisson generalized linear models (GLMs) with a log-link function to compare secondary outcomes, an offset of time and robust standard errors to compare rates of dual method use and unmet need for FP between arms accounting for clustering (Table 4). This analytical approach is appropriate for rare outcomes.([88](#_ENREF_88), [89](#_ENREF_89)) We will conduct an exploratory analysis comparing VL suppression (<1000 copies/mL) between arms among women who become pregnant using generalized estimating equations with a log link, exchangeable correlation structure, and clustering by clinic. Additional exploratory analysis comparing unintended pregnancy rates between arms will be done using a Poisson GLM as described above. Data will be disaggregated by age, marital status, fertility plans, FP method and user type (initiator, continuer, or switcher). Although we expect low attrition and no differences in attrition by arms we will assess whether there is selection bias due to attrition by comparing women retained vs. not retained and report any differences. We will use multivariate imputation by chained equations to impute variables with >25% missing values.

**Table 4. cRCT outcomes**

| **Outcome** | **Outcome type** | **Indicator** | **Source** | **Timing of assessment** | **Statistical Analysis** |
| --- | --- | --- | --- | --- | --- |
| **Aim 1** | | | | | |
| FP discontinuation | Primary | No FP used at exit (among FP users who desire pregnancy prevention) | Survey, clinic records | Quarterly, by 2 years | Cox proportional hazards, time to first event |
| Uptake of dual methods | Secondary | Using condoms and another modern FP method | Survey, clinic records | Quarterly, by 2 years | Poisson GLM (log-link with robust SE |
| Unmet need for FP | Secondary | Desire for pregnancy prevention but not using FP | Survey, clinic records | Quarterly, at 2 years | Poisson GLM (log-link with robust SE) |
| VL suppression at conception | Exploratory/  modeled | VL<1000 copies/mL among women planning pregnancy/pregnant | Lab results | First clinic visit when pregnant/planning pregnancy | Poisson GLM (log-link with robust SE) |
| Unintended pregnancy | Exploratory/  modeled | Pregnancy among women not intending pregnancy (excludes ambivalence) | Survey, clinic records, model | By 2 years | Poisson GEE (log-link with robust SE) |

## PRE-RCT WORKSHOPS (pre-work AIM 1)

**12.1.i Pre-trial formative workshop** We will conduct a workshop among key stakeholders including providers, health care leaders from central and county MOH as well as WLWH receiving HIV care. We will purposefully recruit health care leaders from central and county MOH who represent both HIV and reproductive health decision makers (including those involved with care integration). We anticipate that each county will have 1-2 county district health officers (n=2-4). In addition, we will recruit family planning providers and clinic leadership from representative clinics (5 from Western and 5 from Nairobi). We will purposefully recruit WLWH with a range of RH experiences (FP continuer, FP initiator, FP switcher, FP discontinuer, intended pregnancy, and pregnancy ambivalence) This workshop will inform SMS development, different user ‘tracks’ which depend on women’s reproductive life plans. This type of qualitative data on HIV/SRH topics have previously been shown to be acceptable in our prior studies, including among WLWH.([48](#_ENREF_48), [68](#_ENREF_68), [69](#_ENREF_69), [76](#_ENREF_76)).

| **Population** | **Providers** | **WLWH** | **Policymakers** |
| --- | --- | --- | --- |
| **Role** | Future end users | Empower Beneficiaries | Shape MW Empower implementation context |
| **Eligibility criteria** | - Age ≥18 - Provider (HIV or FP) at clinic | - HIV-infected - Reproductive age (18-45; 14-17 if emancipated minors) | - Age ≥18 - Member of national or county health department/ reproductive health division |
| **Objectives** | - Optimize counseling strategies - Refine SMS messaging - Define counseling and SMS flow - Refine SMS response & referral protocol/decision guide | | - Define Empower context in policy agenda |

Pre-trial data from the workshop will be directly integrated to support intervention modifications, including optimizing tablet-based counseling strategies, SMS content and flow of the intervention. We will conduct the workshop within the IMB (Information Motivation Behavioral) based conceptual framework (Figure 3) which will help maximize intervention acceptability, feasibility and durability.

## **AIM 2 – In-depth Interviews and Focus Group Discussions (FGDs)** (Acceptability, appropriateness, feasibility, and scalability of Mobile WACh Empower implementation under real-world conditions)

**12.1.ii IDIs and FGDs during the trial (Aim 2)** FGDs will be conducted with WLWH during the trial to get their feedback on the counseling tool. Two FGDs will be conducted, stratified by age, at each intervention site (10 WLWH FGDs in total) – one with young women (<25 year olds) and second with older women (>=25 years old). All FGDs will include 6-10 women per FGD and they will be recruited after enrollment. During the trial, we will also conduct IDIs with healthcare providers to understand how they are using the counseling tool to provide reproductive health counseling to WLWH and gather their inputs to improve the counseling tool. We will also we will ask them to fill a short survey on acceptability (i.e. your perception if the Empower counselling tool is agreeable, palatable, or satisfactory), appropriateness (i.e. perceived fit, relevance, or compatibility of the Empower counselling tool in your current role as a healthcare provider), and feasibility (i.e. the extent to which the Empower counselling tool can be successfully used or carried out within a healthcare facility) of the counseling tool. This information would help in revising the counseling tool and improve its implementation. From each intervention site, we will interview ~3 providers who are currently employed at the intervention facility, provide FP services to WLWH, and who’ve interacted with the counseling tool (~15 IDIs in total).

**Exit FGDs (Aim 2)** FGD recruitment will begin when 50% of women exit the trial. We will conduct 2 FGDs among WLWH exiting the trial at 24 months at intervention sites (10 WLWH FGDs total). FGDs will be stratified by RH outcome (FP continuer, FP switcher, non-pregnant FP discontinuer, pregnant FP discontinuer, pregnancy ambivalence); each site will be randomly assigned to recruit women for 2 of these groups (**Figure 10**). We will purposely recruit women with a range of SMS engagement based on percentiles of SMS responses (≤25, >25-75, and >75%ile) to capture varied user experiences within FGDs. We will also conduct FGDs with HIV providers (1 per intervention site). All FGDs will include 6-10 people per FGD. FGDs will allow participants to describe experiences or situations/issues related to RH needs, in their own words and in a way they would
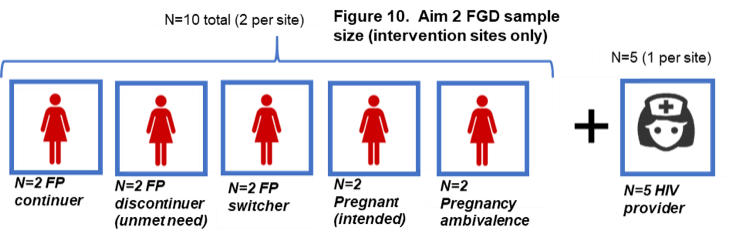
commonly discuss these concepts. FGD sample sizes are intended to capture diversity in experiences, while also reaching thematic saturation; i.e., when no new concepts emerge with subsequent FGDs. However, we will increase the number of FGDs with WLWH if new themes emerge at the end of FGDs. Provider sample size is limited to the number of providers at each intervention site.

| **Table 5. Concepts assessed during FGDs at study end (24 months) (Aim 2)** | |
| --- | --- |
| ***WLWH*** *- Analysis guided by IMB Theory* | ***Providers***  *- Analysis guided by CFIR* |
| Identify community norms on intervention barriers & facilitators   - Perspectives on utility of SMS - How intervention influenced information & motivation informing RH decision-making - Impact on RH behaviors - Intervention preferences, strengths, limitations | - Key influences on intervention implementation - Evaluate context, climate, resources, and intervention components that impact intervention acceptability, feasibility, and appropriateness - Identify strategies for future intervention scalability |

FGD topic guides for WLWH will be grounded in IMB Theory and will evaluate how Mobile WACh Empower influenced information and motivations informing RH decision-making, as well as intervention impact on RH behaviors, including self-efficacy for safe pregnancy/FP use and continuation **(Table 5)**. Guides will assess acceptability through participant preferences, strengths, and limitations of the intervention. Provider FGD guides will be grounded in the LMIC adapted Consolidated Framework for Implementation Research (CFIR).([90](#_ENREF_90), [91](#_ENREF_91)) CFIR is a flexible, meta-theoretical framework designed to describe heterogeneity in implementation across settings, and the relative effect of key constructs in influencing implementation outcomes.([90](#_ENREF_90)) Provider FGD guides will elicit information on individual and health systems influences on acceptability, feasibility and future scalability, and key information about the role of context in influencing implementation.

A Kenyan team trained in qualitative research methods will conduct all IDIs (~1 hour/IDI) and FGDs (~1.5-2 hours/FGD) in English, Kiswahili, or Dhuluo, depending on participant preferences. IDIs and FGDs will be audio recorded, transcribed, and translated into English if necessary. Following IDIs and FGDs, the researcher that led the discussion will complete a targeted debrief report to share overall IDI/FGD impressions and preliminary content discussed with investigators in real time.([92](#_ENREF_92))

**12.1.iii IDI and FGD analysis procedures**

We will analyze transcripts using directed content analysis([93](#_ENREF_93)) and ATLAS.ti. Dr. Beima-Sofie will lead the research team to develop a preliminary codebook based on specific IMB theory or CFIR constructs, and additional themes identified in debriefing reports. The codebook will be reviewed against full length transcripts and iteratively revised until all key themes are captured in the coding structure. After completing a consensus coding process and ensuring consistent application and interpretation of codes, all transcripts will be coded by 2 team members. Based on IMB theory (WLWH transcripts) or CFIR (provider transcripts), code co-occurrence tables and queries will be used to identify key influences on user acceptability, appropriateness, feasibility and future scalability of Mobile WACh Empower. Thematic network analysis will be used to group identified influences into larger thematic categories and describe the network of related concepts influencing user experiences and health systems considerations, and possible strategies to overcome or harness identified barriers and facilitators. **Findings from the IDIs, FGDs and surveys to assess RH outcomes will be integrated into future designs to 1) better understand causality and mechanisms of action for specific outcomes and 2) adapt the intervention and Mobile WACh Empower platform for scale, including refinement of SMS messages for this environment, population, and specific outcomes, as well as integration within existing workflows.**

## Aim 3- Health economic analysis and mathematical model

Micro-costing and time-motion observation studies will be conducted to estimate the incremental cost of implementing Mobile WACh Empower from a health system’s perspective. We will collect trial costs from facilities utilizing standardized cost menus. Costs will be divided into categories (start-up, training, clinic space, human resources, supplies and equipment). Resource use will be assessed by examining study budgets, expense reports, and conducting study staff interviews. Capital and start-up costs will be annualized assuming a useful life of 5 years and discounting at 3% following standard guidelines.([94](#_ENREF_94)) We will conduct time-motion observation of intervention activities for up to 50 patient/provider interactions to measure provider time needed for counseling in intervention and control facilities, and staff time to operate and respond to Mobile WACh SMS. Research time (e.g., informed consent) and supplies will be excluded. Costs of labor and FP commodities will be measured in the trial. We will use the contraceptive method mix observed in Mobile WACh Empower, and micro-costing data, to estimate average cost per FP user, updating a historical model on costs of FP on PMTCT.([95](#_ENREF_95))

**MTCT Model** We will adapt an existing MTCT Markov model (*Meisner/Drake review Int AIDS Soc 2021*)([64](#_ENREF_64)), including upstream elements related to pregnancy risk and intentions, and economic impact of Mobile WACh Empower (including intervention costs incurred/averted). Current model parameters include time since HIV diagnosis, ART use/adherence, infant ARV use, viral suppression, and breastfeeding. We will estimate costs of ART provision and other healthcare expenses from the literature. We will adapt our model to incorporate estimates of unintended pregnancies following prior methods*(*[*95*](#_ENREF_95)*)*, including: unmet need for FP among WLWH (from Mobile WACh Empower trial), current maternal ART and infant ARV regimens, and contraceptive failure rates from developing countries. ([96](#_ENREF_96)) Reduction in births among WLWH who desire pregnancy prevention (need for FP) will be calculated based on the ratio of the contraceptive index (1-1.08x contraceptive prevalence rate [CPR] x efficacy) for women who receive the intervention vs SOC (current CPR and unmet need).([95](#_ENREF_95)) We will calibrate the model to estimates of unintended pregnancy reported by Track 20 and MTCT rates in Kenya. We will project intervention impact on unintended pregnancies and MTCT, calculating incremental cost-effectiveness ratios (ICERs) as the ratio of costs divided by the difference in health outcomes (unintended pregnancy and infant HIV) of the intervention compared to the SOC over a 20 year time horizon. Consistent with guidelines, we will discount costs and health benefits at 3% annually; ICERs below Kenya’s per capita gross domestic product will be considered cost-effective([97](#_ENREF_97)). We will conduct sensitivity analyses to identify influential assumptions.

## 12.7 Study Materials

Equipment: The grant award includes support for SMS platform messaging delivery and receipt, 6 tablets, field office supplies (stationary, paper, toner), 6 laptop computers.

Personnel: The grant award includes support for KNH, UW and MOH investigators, clinic personnel, the data team, and two study coordinators (clinical coordinator (Nairobi based) and overall coordination (Seattle based)). Study personnel working in Kenya will be hired through KNH according to standard procedures.

## 12.8 Training Procedures

Dr. John Kinuthia, Dr. Alison Drake, Dr Jennifer will supervise training of clinical personnel in study procedures. This will include research ethics, neonatal health and FP counseling and completion of surveys.

##

## 12.9 Quality Assurance Procedures

Clinical care: No clinical care will be provided by study staff. However, SMS and referral procedures will be developed in accordance with Government of Kenya guidelines for the care of WLWH and reproductive health and family planning.  Data collected as part of the study will be abstracted from clinic medical records.

Adherence to protocol: Weekly reporting of enrolment, follow-up, medical complications and results will enable us to monitor that the study is running according to approved protocols.  Frequent reporting will also enable us to quickly respond to any problems that arise during the study.

# 13. ETHICAL CONSIDERATIONS

## 13.1 Consent explanation

Please see the attached consent forms:

Consent 1: RCT study participation

Consent 2: Pre-RCT Workshop discussion

Consent 3: Post-RCT FGD women

Consent 4: FGD with women

Consent 5: IDI with provider

## 13.2 Institutional Review Board

This is a collaborative research proposal that will involve field procedures in Nairobi and data analyses in Nairobi and Seattle. The study will be reviewed by the Kenyatta National Hospital/University of Nairobi (KNH/UON) Ethics and Research Committee (ERC), and the University of Washington Institutional Review Board (IRB). The study will not recruit subjects prior to approval from both the UW IRB and the KNH/UON ERC.

## 13.3 Risks to subjects

**Physical:** The study involves no medical interventions therefore we anticipate no risk of serious harm to participants.

**Other:** SMS: There is a potential risk of disclosure of an individual’s person information to others in situations where phones are shared or stolen. We will minimize these risks through counseling in the informed consent process and ensuring women understand the type of messaging that will occur.

Access to clinical records: There could be a breach of confidentiality in the process of retrieving participants’ medical records. This will be mitigated by training all study staff on data management and storage to ensure confidentiality of sensitive data is maintained.

Loss of Confidentiality in Workshop and FGD: Women will be notified that by participating in the Workshop and FGD, loss of confidentiality is a possible risk of participating. They will be informed that this is very unlikely as every measure will be taken to ensure confidentiality.

Alternative treatments or procedures: Not applicable

## 13.4 Protection against risks

Informed consent: Study staff will give potential participants verbal and written information about the study. Study staff will go through the consent form and will explain to each potential participant the purpose, risks and benefits of the study in the language of the participant’s choosing. Participants will be informed that they can choose to withdraw from the study at any point and for any reason. They will also be informed that participation is completely voluntary. Participants will have a chance to ask questions about the study and offered participation. Oral consent will be obtained at time of enrollment. We will obtain electronic written informed consent for study participation into the RCT. Study staff will go through the consent form and will explain to each potential participant the purpose, risks and benefits of the study in the language of the participant’s choosing. Participants will be informed that they can choose to withdraw from the study at any point and for any reason. They will also be informed that participation is completely voluntary. Participants will be offered a copy of the consent form to keep. Participants who agree to participate in study activities will sign the informed consent form electronically in the presence of the study nurse using REDCap’s signature field in which a participant will use a finger or stylus to mark their signature on the study tablet. The electronic signatures will be saved on a secure REDCap server (managed by the University of Washington Institute of Translational Health Sciences), separate from study data. The electronic signature is not a digital signature. Electronic collection of consent signatures allows for real time consent quality monitoring, as well as secure, encrypted, storage of participant names and signatures. Before signing, the study staff will explain to participants that signing the form electronically will serve as the equivalent of signing a physical document. Study staff will help participants navigate the electronic signature and consent process. Once a participant electronically signs, the study staff will ask the participant to review the inputted information to certify that it is correct, before submitting to the server. Study staff will have access to the technology required for electronic consenting. Consenting will be conducted face-to-face if possible, according to Kenyan COVID-19 precaution guidelines, but may be conducted over the phone to adhere to social distancing from KNH and UW ethical research guidelines. Study staff will have a dedicated phone available for client use if clients do not have a personal phone that can be used. These phones will not hold any participant information and will be sanitized between uses. If a participant cannot read or has trouble reading, study staff will read the form to participants. Study staff will also review the form with all participants after they have finished reading it to ensure participant understanding. Participants who cannot read or write will be able to provide documentation of writing consent by making a mark, such as an ‘X’ on the subject signature line after hearing the consent form read to them in the presence of a witness who signs and dates the consent form. The witness will be impartial (not a member of the research team).

SMS: There is a potential risk of disclosure of an individual’s person information to others in situations where phones are shared or stolen. However, we will not send any sensitive information via text. Study staff will specifically demonstrate example messages, to ensure potential cohort study participants agree to receipt of this information. Participants will only be enrolled once they understand study procedures and find the messaging acceptable.

Access to clinical records: There could be a breach of confidentiality in the process of retrieving participants’ medical records. This will be mitigated by training all study staff on data management and storage to ensure confidentiality of sensitive data is maintained. Databases will not include patient identifiers and will be encrypted and password protected.

Loss of confidentiality in workshops and FGDs: In order to protect participant confidentiality, participants will be identified in audio recordings and transcripts only by their study ID, not their name. Workshop and FGD participants will be counselled on the importance of not sharing the identity of other participants or details regarding the content of the discussion with anyone outside the group.

DSMB: We will establish a DSMB who will regularly monitor protocol implementation, adverse events and study outcomes.

Undiagnosed conditions: It is possible we will detect previously undiagnosed conditions such as depression and intimate partner violence. Participants will be referred for appropriate health and social services.

SMS withdrawal: Participants in the intervention group may withdraw from receiving SMS messages at any time. Study staff will complete an SMS Withdrawal form for any participants who wish to withdraw from the system. Participants who withdraw from SMS can continue or withdraw from the study.

Study withdrawal: Participants may withdraw from the study at any time for any reason. Study staff will complete a Study Termination form for any participants choosing to terminate early. All data collection and SMS messaging will discontinue at that point.

## 13.5 Potential benefits

The study will contribute to our understanding of how to deliver education, counseling and support for reproductive life planning in WLWH. It will also provide information that can be used to improve services to WLWH. It may help more WLWH achieve their family planning goals. The study will also shape future counseling programs. Participants in the intervention arm may personally benefit from having access to advice about reproductive health.

## 13.6 Compensation

A nominal travel reimbursement will be provided for participant travel to the study clinic during the 2 year study visit period. Accommodation, transport, per diem and other associated incidentals equivalent to standard rates will be reimbursed for workshop participation.

## 12.7 Importance of the knowledge to be gained

This study will determine the effectiveness and health system impact of a counseling tool and 2-way SMS communication between WLWH and nurses to improve reproductive health outcomes. This pragmatic trial will generate actionable data about a scalable intervention that has the potential to improve reproductive health outcomes among WLWH.

# 14. DATA MANAGEMENT

14.1 FGDs and IDIs

FGD and IDI audio recordings and transcripts will be uploaded to a password-protected computer and erased from the recorder within one day of interview. Recordings will be erased once transcripts have been validated, no more than 6 months after generation of the recording.

14.2 Questionnaires

All questionnaires will be administered using the tablet-based REDCap platform. Tablets will be password-protected and questionnaires will be transmitted to a secure server daily and erased from the tablets. Data will be transmitted via secure socket layer (SSL) and only accessible by authenticated users. Tablets will be stored in a secure, locked office accessible to study staff only. All participants will be assigned a non-identifiable study ID number upon enrolment. All data records will be identified by study ID only. The link between identifiable participant information and study IDs will be locked in a secure, locked location and destroyed following study completion. Study analysts will receive only coded data.

14.3 Electronic Records

Consent forms and participant locator information will be stored electronically in password protected tablets. They will then be transmitted to a secure server daily and erased from the tablets. They will be transmitted via secure socket layer (SSL) and only accessible by authenticated users.

14.4 Data Ownership

The proposed project is a collaborative effort between investigators at the UW and KNH. The aforementioned institutions will jointly share ownership of the data. Study investigators at the UW KNH and WIH will have full access to the data. Authorship on publications, conference presentations, abstracts and other materials generated from this study will reflect contribution to design, execution and analysis of the study.

14.5 Data Release/Sharing Policy

This study will comply with the [NIH Public Access Policy](http://publicaccess.nih.gov/policy.htm), which ensures that the public has access to the published results of NIH funded research. It requires scientists to submit final peer-reviewed journal manuscripts that arise from NIH funds to the digital archive [PubMed Central](http://www.pubmedcentral.nih.gov/) upon acceptance for publication.

# 15. RESULTS DISSEMINATION

We will establish a community advisory board for the study. This will include medical providers and community members living in the study area.  We will disseminate results to this board twice a year and ask for guidance if community issues arise.

Study findings will be shared with the participating facilities, County leadership and Kenyan Ministry of Health at its conclusion as a presentation or written report. Findings will be disseminated to the research community in the form of conference presentations and journal articles.

# 16. STUDY LIMITATIONS

*The intervention will not address all structural barriers to RH,* such as availability of skilled providers; but barriers are expected to be equally distributed by arm and intervention counseling may indirectly alleviate provider-related barriers to RH counseling. Self-reported RH outcomes are subject to *social desirability bias*. To minimize bias we will collect medical record and pharmacy data on FP, pregnancy, and VL. *Threats to internal validity* may exist if temporal changes impact study outcomes; the cRCT design mitigates threats. We will document programmatic changes (i.e., personnel, supplies, stock-outs) and statistically adjust for changes over time if necessary. The *counseling tool relies on batteries*, but tablets last 1-2 days with typical use (6-8 consistent hours) without charging and we will supply back-up batteries. This approach is feasible to implement if the intervention is scaled. If smartphone coverage increases, counseling content could be incorporated into applications designed for use on personal mobile devices outside of clinic settings in future studies. Due to the low incidence of pregnancy (10/100 person-years), *we lack power to determine impact on unintended pregnancies and MTCT in the trial*. We will use mathematical modeling to measure projected health and economic impact of the intervention on these outcomes on a national scale, and provide important updates of cost-effectiveness on reducing on unmet need for FP among WLWH.

# 16. STUDY TIMELINE

We anticipate that this study will take 5 years to complete. In the first year, we will apply for and obtain ethical approval from institutional review boards (IRBs) at the University of Washington and Kenyatta National Hospital. We anticipate receiving IRB approval by Year 1 Month 4.

For Aim 1, we will conduct focus group discussions (FGDs) with women and providers in Year 1 Months 5-6; data from these discussions will be used to make adaptations to the Mobile WACh platform, including tailoring SMS content for women living with HIV (WLWH) and incorporating key relevant variables identified from the counseling tool. FGDs will be analyzed during Year 1 Q3. We will simultaneously develop trial standard operating procedures (SOPs) and case report forms (CRFs) for data collection during the first 6 months of Year 1. By the end of Year 1, we anticipate completing the adaptations and extensive user testing, initiating training and field site preparation activities including community sensitization and outreach. In Year 2, we will complete training, and begin recruiting and enrolling trial participants. Women will be followed for two years with visits conducted between Q2 Year 2 and Q1 Year 5. We will continually produce reports, clean, and analyze data throughout the trial to promote rapid completion of data analysis at the end of the trial. Final data cleaning, analysis, and manuscript preparation and submission will occur in Year 5.

For Aim 2, IDIs with providers and FGDs with women will be conducted during the trial period. Exit FGDs with women and HIV care providers will occur Year 4 in Q3 and Q4. FGDs will be analyzed immediately following the FGDs, with analysis complete by Year 5 Q1. Manuscript development from FGD findings will begin in Year 4 Q4.

For Aim 3, micro-costing will occur in Year 2 and Year 3 Q1, time-motion studies from Year 3 through Year 4 Q2, and mathematical model development beginning in Year 4. Dissemination of results (including manuscript submissions) will occur during Year 4 Q4 and the last 6 months of Year 5.

At the end of the study, we will present findings to stakeholders at facilities and MOH, including results from exit FGDs, which will support intervention implementation and scale-up.

|  | Year 1 | | | | Year 2 | | | | Year 3 | | | | Year 4 | | | | Year 5 | | | |
| --- | --- | --- | --- | --- | --- | --- | --- | --- | --- | --- | --- | --- | --- | --- | --- | --- | --- | --- | --- | --- |
|  | Q1 | Q2 | Q3 | Q4 | Q1 | Q2 | Q3 | Q4 | Q1 | Q2 | Q3 | Q4 | Q1 | Q2 | Q3 | Q4 | Q1 | Q2 | Q3 | Q4 |
| ***Aim 1 cRCT*** | | | | | | | | | | | | | | | | | | | | |
| Ethical approvals |  |  |  |  |  |  |  |  |  |  |  |  |  |  |  |  |  |  |  |  |
| Structured workshops to guide adaptions to Mobile WACh platform/tailor for WLWH |  |  |  |  |  |  |  |  |  |  |  |  |  |  |  |  |  |  |  |  |
| Analyze Workshop discussions |  |  |  |  |  |  |  |  |  |  |  |  |  |  |  |  |  |  |  |  |
| Develop SOPs and CRFs |  |  |  |  |  |  |  |  |  |  |  |  |  |  |  |  |  |  |  |  |
| Adapt Mobile WACh Platform |  |  |  |  |  |  |  |  |  |  |  |  |  |  |  |  |  |  |  |  |
| Training |  |  |  |  |  |  |  |  |  |  |  |  |  |  |  |  |  |  |  |  |
| Enrollment |  |  |  |  |  |  |  |  |  |  |  |  |  |  |  |  |  |  |  |  |
| Follow-up |  |  |  |  |  |  |  |  |  |  |  |  |  |  |  |  |  |  |  |  |
| Analysis and manuscript development |  |  |  |  |  |  |  |  |  |  |  |  |  |  |  |  |  |  |  |  |
| ***Aim 2 Implementation evaluation*** | | | | | | | | | | | | | | | | | | | | |
| Develop SOPs and FDG guides |  |  |  |  |  |  |  |  |  |  |  |  |  |  |  |  |  |  |  |  |
| Conduct FGDs |  |  |  |  |  |  |  |  |  |  |  |  |  |  |  |  |  |  |  |  |
| Analyze FGDs |  |  |  |  |  |  |  |  |  |  |  |  |  |  |  |  |  |  |  |  |
| Analysis and manuscript development |  |  |  |  |  |  |  |  |  |  |  |  |  |  |  |  |  |  |  |  |
| ***Aim 3 Health and economic analysis*** | | | | | | | | | | | | | | | | | | | | |
| Micro-costing |  |  |  |  |  |  |  |  |  |  |  |  |  |  |  |  |  |  |  |  |
| Time-motion studies |  |  |  |  |  |  |  |  |  |  |  |  |  |  |  |  |  |  |  |  |
| Mathematical model development |  |  |  |  |  |  |  |  |  |  |  |  |  |  |  |  |  |  |  |  |
| Analysis and manuscript development |  |  |  |  |  |  |  |  |  |  |  |  |  |  |  |  |  |  |  |  |
| ***Present to stakeholders*** |  |  |  |  |  |  |  |  |  |  |  |  |  |  |  |  |  |  |  |  |

# 17. REFERENCES

1. Chen Y, Begnel E, Muthigani W, Achwoka D, McGrath CJ, Singa C, Gondi J, Ng'ang'a L, Langat A, John- Stewart G, Kinuthia J, Drake AL. Higher Contraceptive Uptake in HIV Treatment Centers Offering Integrated Family Planning Services: a National Survey in Kenya. Contraception. 2020; 102(1):39-45. PubMed PMID: 32298715.
2. Grossman D, Onono M, Newmann SJ, Blat C, Bukusi EA, Shade SB, Steinfeld RL, Cohen CR. Integration of family planning services into HIV care and treatment in Kenya: a cluster-randomized trial. AIDS. 2013;27 Suppl 1:S77-85. Epub 2013/10/23. doi: 10.1097/QAD.0000000000000035. PubMed PMID: 24088687.
3. Kosgei RJ, Lubano KM, Shen C, Wools-Kaloustian KK, Musick BS, Siika AM, Mabeya H, Carter EJ, Mwangi A, Kiarie J. Impact of integrated family planning and HIV care services on contraceptive use and pregnancy outcomes: a retrospective cohort study. J Acquir Immune Defic Syndr. 2011;58(5):e121-6. Epub 2011/10/04. doi: 10.1097/QAI.0b013e318237ca80. PubMed PMID: 21963940; PMCID: PMC3779789.
4. Wilcher R, Hoke T, Adamchak SE, Cates W, Jr. Integration of family planning into HIV services: a synthesis of recent evidence. AIDS. 2013;27 Suppl 1:S65-75. Epub 2013/10/23. doi: 10.1097/QAD.0000000000000051. PubMed PMID: 24088686.
5. Mutemwa R, Mayhew S, Colombini M, Busza J, Kivunaga J, Ndwiga C. Experiences of health care providers with integrated HIV and reproductive health services in Kenya: a qualitative study. BMC Health Serv Res. 2013;13:18. Epub 2013/01/15. doi: 10.1186/1472-6963-13-18. PubMed PMID: 23311431; PMCID: PMC3599716.
6. Smit JA, Church K, Milford C, Harrison AD, Beksinska ME. Key informant perspectives on policy- and service- level challenges and opportunities for delivering integrated sexual and reproductive health and HIV care in South Africa. BMC Health Serv Res. 2012;12:48. Epub 2012/03/01. doi: 10.1186/1472-6963-12-48. PubMed PMID: 22369041; PMCID: PMC3311559.
7. Todd CS, Anderman TC, Long S, Myer L, Bekker LG, Petro GA, Jones HE. A systematic review of contraceptive continuation among women living with HIV. Contraception. 2018;98(1):8-24. Epub 2018/02/13. doi: 10.1016/j.contraception.2018.02.002. PubMed PMID: 29432719.
8. Schivone GB, Glish LL. Contraceptive counseling for continuation and satisfaction. Curr Opin Obstet Gynecol. 2017;29(6):443-8. Epub 2017/09/25. doi: 10.1097/GCO.0000000000000408. PubMed PMID: 28938374.
9. Mangone ER, Lebrun V, Muessig KE. Mobile Phone Apps for the Prevention of Unintended Pregnancy: A Systematic Review and Content Analysis. JMIR Mhealth Uhealth. 2016;4(1):e6. Epub 2016/01/21. doi: 10.2196/mhealth.4846. PubMed PMID: 26787311; PMCID: PMC4738182.
10. Haberlen SA, Narasimhan M, Beres LK, Kennedy CE. Integration of Family Planning Services into HIV Care and Treatment Services: A Systematic Review. Stud Fam Plann. 2017;48(2):153-77. Epub 2017/03/25. doi: 10.1111/sifp.12018. PubMed PMID: 28337766; PMCID: PMC5516228.
11. Castano PM, Bynum JY, Andres R, Lara M, Westhoff C. Effect of daily text messages on oral contraceptive continuation: a randomized controlled trial. Obstet Gynecol. 2012;119(1):14-20. Epub 2011/12/07. doi: 10.1097/AOG.0b013e31823d4167. PubMed PMID: 22143257.
12. Canto De Cetina TE, Canto P, Ordonez Luna M. Effect of counseling to improve compliance in Mexican women receiving depot-medroxyprogesterone acetate. Contraception. 2001;63(3):143-6. Epub 2001/05/23. doi: 10.1016/s0010-7824(01)00181-0. PubMed PMID: 11368986.
13. Women and girls and HIV. Geneva: 2018. Deglise C, Suggs LS, Odermatt P. SMS for disease control in developing countries: a systematic review of mobile health applications. J Telemed Telecare. 2012;18(5):273-81. Epub 2012/07/25. doi: 10.1258/jtt.2012.110810. PubMed PMID: 22826375.
14. Ahmed S, Li Q, Liu L, Tsui AO. Maternal deaths averted by contraceptive use: an analysis of 172 countries. Lancet. 2012;380(9837):111-25. Epub 2012/07/13. doi: 10.1016/S0140-6736(12)60478-4. PubMed PMID: 22784531.
15. WHO UNICEF. Geneva: 2007. Guidance on global scale-up of the prevention of mother to child transmission of HIV: Towards universal access for women, infants, and young children and eliminating HIV and AIDS among children.
16. Cleland J, Bernstein S, Ezeh A, Faundes A, Glasier A, Innis J. Family planning: the unfinished agenda. Lancet. 2006;368(9549):1810-27. doi: 10.1016/S0140-6736(06)69480-4. PubMed PMID: 17113431.
17. Trivedi D. Cochrane review summary: Education for contraceptive use by women after childbirth. Primary health care research & development. 2013;14(2):109-12. doi: 10.1017/S1463423613000091. PubMed PMID: 23461873.
18. Lopez LM, Hiller JE, Grimes DA, Chen M. Education for contraceptive use by women after childbirth. The Cochrane database of systematic reviews. 2012;8:CD001863. doi: 10.1002/14651858.CD001863.pub3. PubMed PMID: 22895923.
19. Patel RC, Morroni C, Scarsi KK, Sripipatana T, Kiarie J, Cohen CR. Concomitant contraceptive implant and efavirenz use in women living with HIV: perspectives on current evidence and policy implications for family planning and HIV treatment guidelines. J Int AIDS Soc. 2017;20(1):21396. Epub 2017/05/23. doi: 10.7448/IAS.20.1.21396. PubMed PMID: 28530033; PMCID: PMC5515020.

1. Patel RC, Onono M, Gandhi M, Blat C, Hagey J, Shade SB, Vittinghoff E, Bukusi EA, Newmann SJ, Cohen CR. Pregnancy rates in HIV-positive women using contraceptives and efavirenz-based or nevirapine-based antiretroviral therapy in Kenya: a retrospective cohort study. Lancet HIV. 2015;2(11):e474-82. Epub 2015/11/02. doi: 10.1016/S2352-3018(15)00184-8. PubMed PMID: 26520927; PMCID: PMC4632202.
2. Patel RC, Stalter RM, Thomas KK, Tamraz B, Blue SW, Erikson DW, Kim CJ, Kelly EJ, Nanda K, Kourtis AP, Lingappa JR, Mugo N, Baeten JM, Scarsi KK, Partners Pr EPST. A pharmacokinetic and pharmacogenetic evaluation of contraceptive implants and antiretroviral therapy among women in Kenya and Uganda. AIDS. 2019;33(13):1995-2004. Epub 2019/07/16. doi: 10.1097/QAD.0000000000002308. PubMed PMID: 31306173; PMCID: PMC6774811.
3. Festin MP, Kiarie J, Solo J, Spieler J, Malarcher S, Van Look PF, Temmerman M. Moving towards the goals of FP2020 - classifying contraceptives. Contraception. 2016;94(4):289-94. Epub 2016/06/12. doi: 10.1016/j.contraception.2016.05.015. PubMed PMID: 27287693; PMCID: PMC5032916. Tessema GA, Streak Gomersall J, Mahmood MA, Laurence CO. Factors Determining Quality of Care in Family Planning Services in Africa: A Systematic Review of Mixed Evidence. PLoS One. 2016;11(11):e0165627. Epub 2016/11/05. doi: 10.1371/journal.pone.0165627. PubMed PMID: 27812124; PMCID: PMC5094662.
4. Noguchi LM, Simelela PN. How should we listen to ECHO? Lancet. 2019;394(10195):275-6. Epub 2019/06/18. doi: 10.1016/S0140-6736(19)31387-X. PubMed PMID: 31204113.
5. Stephenson R, Beke A, Tshibangu D. Contextual influences on contraceptive use in the Eastern Cape, South Africa. Health Place. 2008;14(4):841-52. doi: 10.1016/j.healthplace.2008.01.005. PubMed PMID: 18308611.
6. Kawale P, Mindry D, Phoya A, Jansen P, Hoffman RM. Provider attitudes about childbearing and knowledge of safer conception at two HIV clinics in Malawi. Reprod Health. 2015;12:17. Epub 2015/03/17. doi: 10.1186/s12978-015-0004-0. PubMed PMID: 25771719; PMCID: PMC4355153.
7. Coll AS, Potter JE, Chakhtoura N, Alcaide ML, Cook R, Jones DL. Providers' perspectives on preconception counseling and safer conception for HIV-infected women. AIDS Care. 2016;28(4):513-8. Epub 2015/11/19. doi: 10.1080/09540121.2015.1112349. PubMed PMID: 26577664; PMCID: PMC4764433.
8. Wanyenze RK, Wagner GJ, Tumwesigye NM, Nannyonga M, Wabwire-Mangen F, Kamya MR. Fertility and contraceptive decision-making and support for HIV infected individuals: client and provider experiences and perceptions at two HIV clinics in Uganda. BMC Public Health. 2013;13:98. Epub 2013/02/05. doi: 10.1186/1471- 2458-13-98. PubMed PMID: 23374175; PMCID: PMC3568663.
9. Spaulding AB, Brickley DB, Kennedy C, Almers L, Packel L, Mirjahangir J, Kennedy G, Collins L, Osborne K, Mbizvo M. Linking family planning with HIV/AIDS interventions: a systematic review of the evidence. AIDS. 2009;23 Suppl 1:S79-88. Epub 2010/02/02. doi: 10.1097/01.aids.0000363780.42956.ff. PubMed PMID: 20081392.
10. Mutisya R, Wambua J, Nyachae P, Kamau M, Karnad SR, Kabue M. Strengthening integration of family planning with HIV/AIDS and other services: experience from three Kenyan cities. Reprod Health. 2019;16(Suppl 1):62. Epub 2019/05/30. doi: 10.1186/s12978-019-0715-8. PubMed PMID: 31138271; PMCID: PMC6538540.
11. McGinn EK, Irani L. Provider-Initiated Family Planning Within HIV Services in Malawi: Did Policy Make It into Practice? Glob Health Sci Pract. 2019;7(4):540-50. Epub 2019/12/04. doi: 10.9745/GHSP-D-19-00192. PubMed PMID: 31791976; PMCID: PMC6927829.
12. Lince-Deroche N, Hendrickson C, Moolla A, Kgowedi S, Mulongo M. Provider perspectives on contraceptive service delivery: findings from a qualitative study in Johannesburg, South Africa. BMC Health Serv Res. 2020;20(1):128. Epub 2020/02/23. doi: 10.1186/s12913-020-4900-9. PubMed PMID: 32085756; PMCID: PMC7035764.
13. Mason J, Medley A, Yeiser S, Nightingale VR, Mani N, Sripipatana T, Abutu A, Johnston B, Watts DH. The role of family planning in achieving safe pregnancy for serodiscordant couples: commentary from the United States government's interagency task force on family planning and HIV service integration. J Int AIDS Soc. 2017;20(Suppl 1):21312. Epub 2017/04/01. doi: 10.7448/IAS.20.2.21312. PubMed PMID: 28361500; PMCID: PMC5461116.
14. Dehlendorf C, Fitzpatrick J, Fox E, Holt K, Vittinghoff E, Reed R, Campora MP, Sokoloff A, Kuppermann M. Cluster randomized trial of a patient-centered contraceptive decision support tool, My Birth Control. Am J Obstet Gynecol. 2019;220(6):565 e1- e12. Epub 2019/02/15. doi: 10.1016/j.ajog.2019.02.015. PubMed PMID: 30763545.
15. Callegari LS, Tartaglione EV, Magnusson SL, Nelson KM, Arteburn DE, Szarka J, Zephyrin L, Borrero S. Understanding Women Veterans' Family Planning Counseling Experiences and Preferences to Inform Patient- Centered Care. Womens Health Issues. 2019;29(3):283-9. Epub 2019/04/15. doi: 10.1016/j.whi.2019.03.002. PubMed PMID: 30981559.
16. Kenya National Bureau of Statistics, Kenya Ministry of Health, Kenya National AIDS Control Council, Kenya Medical Research Institute, and Kenya National Council for Population and Development. Kenya Demographic and Health Survey 20142015.
17. Chamie G, Kwarisiima D, Clark T, Kabami J, Jain V, Geng E, Petersen M, Thirumurthy H, Kamya M, Havlir DV, Charlebois E. Integrated community HIV testing campaigns: Leveraging HIV infrastructure for non- communicable diseases. Journal of the International AIDS Society. 2012;15:236.
18. Kim YM, Davila C, Tellez C, Kols A. Evaluation of the World Health Organization's family planning decision- making tool: improving health communication in Nicaragua. Patient education and counseling. 2007;66(2):235- 42. doi: 10.1016/j.pec.2006.12.007. PubMed PMID: 17250989.
19. Bakari JP, McKenna S, Myrick A, Mwinga K, Bhat GJ, Allen S. Rapid voluntary testing and counseling for HIV. Acceptability and feasibility in Zambian antenatal care clinics. Annals of the New York Academy of Sciences. 2000;918:64-76. Epub 2000/12/29. PubMed PMID: 11131736.
20. Lester RT, Ritvo P, Mills EJ, Kariri A, Karanja S, Chung MH, Jack W, Habyarimana J, Sadatsafavi M, Najafzadeh M, Marra CA, Estambale B, Ngugi E, Ball TB, Thabane L, Gelmon LJ, Kimani J, Ackers M, Plummer FA. Effects of a mobile phone short message service on antiretroviral treatment adherence in Kenya (WelTel Kenya1): a randomised trial. Lancet. 2010;376(9755):1838-45. Epub 2010/11/13. doi: S0140-6736(10)61997-6 [pii]10.1016/S0140-6736(10)61997-6. PubMed PMID: 21071074.
21. United N. ECOSOC ‘Texting 4 Health campaign'; Jinja, UgandaJune 2009.
22. Joseph-Davey D, Ponce W, Augusto O, Traca D, de Palha de Sousa C. Improved uptake of institutional birth and early infant HIV diagnosis following SMS reminders among PMTCT patients in Mozambique: a randomized control trial. 7th International AIDS Society Conference on HIV Pathogenesis, Treatment and Prevention; June 30 - July 3, 2013; Kuala Lampur, Malaysia. 2013.
23. Harrington EK, Drake AL, Matemo D, Ronen K, Osoti AO, John-Stewart G, Kinuthia J, Unger JA. An mHealth SMS intervention on Postpartum Contraceptive Use Among Women and Couples in Kenya: A Randomized Controlled Trial. Am J Public Health. 2019;109(6):934-41. Epub 2019/05/09. doi: 10.2105/AJPH.2019.305051. PubMed PMID: 31067089; PMCID: PMC6507993.

1. Unger JA, Ronen K, Perrier T, DeRenzi B, Slyker J, Drake AL, Mogaka D, Kinuthia J, John-Stewart G. Short message service communication improves exclusive breastfeeding and early postpartum contraception in a low- to middle-income country setting: a randomised trial. BJOG. 2018;125(12):1620-9. Epub 2018/06/21. doi: 10.1111/1471-0528.15337. PubMed PMID: 29924912; PMCID: PMC6179930.
2. Johnson D, Juras R, Riley P, Chatterji M, Sloane P, Choi SK, Johns B. A randomized controlled trial of the impact of a family planning mHealth service on knowledge and use of contraception. Contraception. 2017;95(1):90-7. Epub 2016/07/17. doi: 10.1016/j.contraception.2016.07.009. PubMed PMID: 27421767.
3. Leon N, Schneider H, Daviaud E. Applying a framework for assessing the health system challenges to scaling up mHealth in South Africa. BMC Med Inform Decis Mak. 2012;12:123. Epub 2012/11/07. doi: 10.1186/1472- 6947-12-123. PubMed PMID: 23126370; PMCID: PMC3534437.
4. Halpern V, Lopez LM, Grimes DA, Stockton LL, Gallo MF. Strategies to improve adherence and acceptability of hormonal methods of contraception. Cochrane Database Syst Rev. 2013(10):CD004317. Epub 2013/10/29. doi: 10.1002/14651858.CD004317.pub4. PubMed PMID: 24163097.
5. Stacey D, Bennett CL, Barry MJ, Col NF, Eden KB, Holmes-Rovner M, Llewellyn-Thomas H, Lyddiatt A, Legare F, Thomson R. Decision aids for people facing health treatment or screening decisions. Cochrane Database Syst Rev. 2011(10):CD001431. Epub 2011/10/07. doi: 10.1002/14651858.CD001431.pub3. PubMed PMID: 21975733.
6. Johnson SL, Kim YM, Church K. Towards client-centered counseling: development and testing of the WHO Decision-Making Tool. Patient education and counseling. 2010;81(3):355-61. doi: 10.1016/j.pec.2010.10.011. PubMed PMID: 21093194.
7. Garnett GP, Cousens S, Hallett TB, Steketee R, Walker N. Mathematical models in the evaluation of health programmes. Lancet. 2011;378(9790):515-25. doi: 10.1016/S0140-6736(10)61505-X. PubMed PMID: 21481448.
8. Brittain K, Phillips TK, Zerbe A, Abrams EJ, Myer L. Long-term effects of unintended pregnancy on antiretroviral therapy outcomes among South African women living with HIV. AIDS. 2019;33(5):885-93. Epub 2019/01/17. doi: 10.1097/QAD.0000000000002139. PubMed PMID: 30649049; PMCID: PMC6528830.
9. Yaya S, Uthman OA, Ekholuenetale M, Bishwajit G. Women empowerment as an enabling factor of contraceptive use in sub-Saharan Africa: a multilevel analysis of cross-sectional surveys of 32 countries. Reprod Health. 2018;15(1):214. Epub 2018/12/24. doi: 10.1186/s12978-018-0658-5. PubMed PMID: 30572927; PMCID: PMC6302468.
10. Corroon M, Speizer IS, Fotso JC, Akiode A, Saad A, Calhoun L, Irani L. The role of gender empowerment on reproductive health outcomes in urban Nigeria. Matern Child Health J. 2014;18(1):307-15. Epub 2013/04/12. doi: 10.1007/s10995-013-1266-1. PubMed PMID: 23576403; PMCID: PMC4022125.
11. What are Global Goods: Digital Square. Available from: ttps://wiki.digitalsquare.io/index.php/What_are_Global_Goods.
12. Riley WT, Rivera DE, Atienza AA, Nilsen W, Allison SM, Mermelstein R. Health behavior models in the age of mobile interventions: are our theories up to the task? Transl Behav Med. 2011;1(1):53-71. Epub 2011/07/29. doi: 10.1007/s13142-011-0021-7. PubMed PMID: 21796270; PMCID: PMC3142960.
13. Fisher WA FJ, Harman J. . The information-motivation-behavioral skills model: A general social psychological approach to understanding and promoting health behavior. . In: Suls J WK, editor. Social Psychological Foundations of Health and Illness Blackwell Publishing Ltd 2003. p. 82-106.

1. Hall KS, Westhoff CL, Castano PM. The impact of an educational text message intervention on young urban women's knowledge of oral contraception. Contraception. 2013;87(4):449-54. Epub 2012/10/16. doi: 10.1016/j.contraception.2012.09.004. PubMed PMID: 23062523; PMCID: PMC3548974.
2. Rajput ZA, Mbugua S, Amadi D, Chepngeno V, Saleem JJ, Anokwa Y, Hartung C, Borriello G, Mamlin BW, Ndege SK, Were MC. Evaluation of an Android-based mHealth system for population surveillance in developing countries. Journal of the American Medical Informatics Association : JAMIA. 2012;19(4):655-9. doi: 10.1136/amiajnl-2011-000476. PubMed PMID: 22366295; PMCID: 3384107.
3. Chen Y, Begnel E, Muthigani W, Achwoka D, McGrath CJ, Singa B, Gondi J, Ng'ang'a L, Langat A, John- Stewart G, Kinuthia J, Drake AL. Higher Contraceptive Uptake in HIV Treatment Centers Offering Integrated Family Planning Services: a National Survey in Kenya. Contraception. 2020. Epub 2020/04/17. doi: 10.1016/j.contraception.2020.04.003. PubMed PMID: 32298715.
4. Rodriguez P, Roberts DA, Meisner J, Sharma M, Newman Owiredu M, Gomez B, Mello MB, Bobrik A, Bodianyk A, Storey A, Githuka G, Chidarikire T, Barnabas RV, Barr-Dichiara M, Jamil MS, Baggaley R, Johnson CC, Taylor MM, Drake AL. Cost-effectiveness of Dual Maternal HIV and Syphilis Testing Strategies in High and Low HIV Prevalence Countries. Lancet Global Health. 2020 (In Press).
5. Policy Brief: Dual HIV/Syphilis Rapid Diagnostic Tests Can be Used as the First Test in Antenatal Care. November 2019. World Health Organization, Geneva; 2019.
6. Dev R, Woods NF, Unger JA, Kinuthia J, Matemo D, Farid S, Begnel ER, Kohler P, Drake AL. Acceptability, feasibility and utility of a Mobile health family planning decision aid for postpartum women in Kenya. Reprod Health. 2019;16(1):97. Epub 2019/07/10. doi: 10.1186/s12978-019-0767-9. PubMed PMID: 31286989; PMCID: PMC6615081.
7. Harrington EK, McCoy EE, Drake AL, Matemo D, John-Stewart G, Kinuthia J, Unger JA. Engaging men in an mHealth approach to support postpartum family planning among couples in Kenya: a qualitative study. Reprod Health. 2019;16(1):17. Epub 2019/02/13. doi: 10.1186/s12978-019-0669-x. PubMed PMID: 30744697; PMCID: PMC6371458.
8. Ronen K, Unger JA, Drake AL, Perrier T, Akinyi P, Osborn L, Matemo D, O'Malley G, Kinuthia J, John- Stewart G. SMS messaging to improve ART adherence: perspectives of pregnant HIV-infected women in Kenya on HIV-related message content. AIDS Care. 2018;30(4):500-5. Epub 2017/12/20. doi: 10.1080/09540121.2017.1417971. PubMed PMID: 29254362; PMCID: PMC5839109.
9. Fairbanks J, Beima-Sofie K, Akinyi P, Matemo D, Unger JA, Kinuthia J, O'Malley G, Drake AL, John-Stewart G, Ronen K. You Will Know That Despite Being HIV Positive You Are Not Alone: Qualitative Study to Inform Content of a Text Messaging Intervention to Improve Prevention of Mother-to-Child HIV Transmission. JMIR Mhealth Uhealth. 2018;6(7):e10671. Epub 2018/07/22. doi: 10.2196/10671. PubMed PMID: 30026177; PMCID: PMC6072973.
10. Perrier T DN, DeRenzi B, Anderson R, Kinuthia J, Unger J, John-Stewart G Engaging Pregnant Women in Kenya with a Hybrid Computer-Human SMS Communication System. CHI '15 Proceedings of the 33rd Annual ACM Conference on Human Factors in Computing Systems; Seoul, Republic of Korea ACM 2015.
11. Unger JA RK, Perrier T, DeRenzi B, Slyker J, Drake A, Mogaka D, Kinuthia J, John-Stewart G. SMS communication improves exclusive breastfeeding and early postpartum contraception in a low to middle income country setting: A randomised trial BJOG. 2018. Epub Aug PMCID: 29924912.
12. Unger JA, Kinuthia J, John-Stewart G. Texting Condolences: Adapting mHealth Programs After Unexpected Pregnancy and Infant Outcomes. JMIR Mhealth Uhealth. 2017;5(12):e176. Epub 2017/12/10. doi: 10.2196/mhealth.8303. PubMed PMID: 29222078; PMCID: PMC5741824.

1. Fairbanks J B-SK, Akinyi P, Matemo D, Unger JA, Kinuthia J, O’Malley G, Drake Al, John-Stewart G, Ronen,

K. You will know that despite being HIV positive you are not alone: Content preferences for an SMS intervention to improve prevention of mother-to-child HIV transmission (PMTCT). JMIR Mhealth Uhealth. 2018(forthcoming

). doi: 10.2196/10671.

1. Drake AL, Unger JA, Ronen K, Matemo D, Perrier T, DeRenzi B, Richardson BA, Kinuthia J, John-Stewart

G. Evaluation of mHealth strategies to optimize adherence and efficacy of Option B+ prevention of mother-to- child HIV transmission: Rationale, design and methods of a 3-armed randomized controlled trial. Contemp Clin Trials. 2017;57:44-50. Epub 2017/03/21. doi: 10.1016/j.cct.2017.03.007. PubMed PMID: 28315480; PMCID: PMC5522580.

1. Harrington EK ME, Drake AL, Matemo D, John-Stewart G, Kinuthia J, Unger JA. “Kindly tell us the truth of that family planning”: men’s and women’s perspectives on a short message service (SMS) approach to improve postpartum family planning education and counseling in Kenya. Contraception 2017;96(4):301. Epub October 2017.
2. Lewis K, Harrington EK, Matemo D, Drake AL, Ronen K, O'Malley G, Kinuthia J, John-Stewart G, Unger JA. Utilizing perspectives from HIV-infected women, male partners and healthcare providers to design family planning SMS in Kenya: a qualitative study. BMC Health Serv Res. 2019;19(1):870. Epub 2019/11/23. doi: 10.1186/s12913-019-4708-7. PubMed PMID: 31752872; PMCID: PMC6873397.
3. Unger JA, Wandika B, Ronen K, Rothschild C, Shih J, Wamalwa D, Muthigani W, Batra M, Kinuthia J, John- Stewart G. Mobile WACh NEO: Engagement of pregnant and postpartum women with a two-way SMS service to improve neonatal outcomes. Pediatric Academic Society,; April 29, 2019; Baltimore, Maryland2019. Brown W, 3rd, Giguere R, Sheinfil A, Ibitoye M, Balan I, Ho T, Brown B, Quispe L, Sukwicha W, Lama JR, Carballo-Dieguez A, Cranston RD. Challenges and solutions implementing an SMS text message-based survey CASI and adherence reminders in an international biomedical HIV PrEP study (MTN 017). J Biomed Inform. 2018;80:78-86. Epub 2018/03/05. doi: 10.1016/j.jbi.2018.02.018. PubMed PMID: 29501908; PMCID: PMC5920551.
4. Perez GM, Hwang B, Bygrave H, Venables E. Designing text-messaging (SMS) in HIV programs: ethics- framed recommendations from the field. Pan Afr Med J. 2015;21:201. Epub 2015/10/01. doi: 10.11604/pamj.2015.21.201.6844. PubMed PMID: 26421096; PMCID: PMC4575710.
5. Lester RT, Mills EJ, Kariri A, Ritvo P, Chung M, Jack W, Habyarimana J, Karanja S, Barasa S, Nguti R, Estambale B, Ngugi E, Ball TB, Thabane L, Kimani J, Gelmon L, Ackers M, Plummer FA. The HAART cell phone adherence trial (WelTel Kenya1): a randomized controlled trial protocol. Trials. 2009;10:87. Epub 2009/09/24. doi: 10.1186/1745-6215-10-87. PubMed PMID: 19772596; PMCID: PMC2760542.
6. Perrier T, Dell N, DeRenzi B, Anderson R, Kinuthia J, Unger JA, John-Stewart G. Engaging pregnant women in Kenya with a hybrid computer-human SMS communication system. Human–Computer Interaction. 2015;In Press.
7. Unger J, Ronen K, Jiang W, Perrier T, Matemo D, Drake AL, Osborn L, Kinuthia J, John-Stewart G. Engagement in bidirectional mobile messaging to support antiretroviral therapy (ART) adherence among peripartum women in Kenya. . 22nd International AIDS Conference (AIDS 2018); 23-27 July 2018; Amsterdam, Netherlands2018.
8. Hsieh HF, Shannon SE. Three approaches to qualitative content analysis. . Qual Health Res 2005;15(9):1277-88.
9. Attride-Sterling J. Thematic Networks: an analystic tool for qualitative research. Qualitative Research. 2001;1:385-405.
10. Kinuthia J, Drake AL, Matemo D, Richardson BA, Zeh C, Osborn L, Overbaugh J, McClelland RS, John- Stewart G. HIV acquisition during pregnancy and postpartum is associated with genital infections and partnership characteristics. AIDS. 2015;29(15):2025-33. Epub 2015/09/10. doi: 10.1097/QAD.0000000000000793. PubMed PMID: 26352880; PMCID: PMC4692052.

1. Beijers R, Jansen J, Riksen-Walraven M, de Weerth C. Maternal prenatal anxiety and stress predict infant illnesses and health complaints. Pediatrics. 2010;126(2):e401-9. Epub 2010/07/21. doi: 10.1542/peds.2009- 3226. PubMed PMID: 20643724.
2. Morgan KE, Forbes AB, Keogh RH, Jairath V, Kahan BC. Choosing appropriate analysis methods for cluster randomised cross-over trials with a binary outcome. Stat Med. 2017;36(2):318-33. Epub 2016/09/30. doi: 10.1002/sim.7137. PubMed PMID: 27680896.
3. Breslow NE. Generalized linear models: checking assumptions and strengthening conclusions. Stat Apl. 1996(8):23-41.
4. Lee J, Chia KS. Estimation of prevalence rate ratios for cross sectional data: an example in occupational epidemiology. Br J Ind Med. 1993;50(9):861-2. Epub 1993/09/01. doi: 10.1136/oem.50.9.861. PubMed PMID: 8398881; PMCID: PMC1061320.
5. Damschroder LJ, Aron DC, Keith RE, Kirsh SR, Alexander JA, Lowery JC. Fostering implementation of health services research findings into practice: a consolidated framework for advancing implementation science. Implement Sci. 2009;4:50. Epub 2009/08/12. doi: 10.1186/1748-5908-4-50. PubMed PMID: 19664226; PMCID: PMC2736161.
6. Means AR, Kemp CG, Gwayi-Chore MC, Gimbel S, Soi C, Sherr K, Wagenaar BH, Wasserheit JN, Weiner BJ. Evaluating and optimizing the consolidated framework for implementation research (CFIR) for use in low- and middle-income countries: a systematic review. Implement Sci. 2020;15(1):17. Epub 2020/03/14. doi: 10.1186/s13012-020-0977-0. PubMed PMID: 32164692; PMCID: PMC7069199.
7. Simoni JM, Beima-Sofie K, Amico KR, Hosek SG, Johnson MO, Mensch BS. Debrief Reports to Expedite the Impact of Qualitative Research: Do They Accurately Capture Data from In-depth Interviews? AIDS and behavior. 2019;23(8):2185-9. Epub 2019/01/23. doi: 10.1007/s10461-018-02387-3. PubMed PMID: 30666522; PMCID: PMC6642848.
8. Hsieh HF, Shannon SE. Three approaches to qualitative content analysis. . Qual Health Res. 2005;15:1277- 88.
9. World Health Organization Statistical Information System: CHOICE (Choosing Interventions that are Cost Effective). Last accessed 2 February 2019 from: h[ttps://www.who.int/choice/cost-effectiveness/en/.](ttps://www.who.int/choice/cost-effectiveness/en/)
10. Halperin DT, Stover J, Reynolds HW. Benefits and costs of expanding access to family planning programs to women living with HIV. AIDS. 2009;23 Suppl 1:S123-30. Epub 2010/02/02. doi: 10.1097/01.aids.0000363785.73450.5a. PubMed PMID: 20081384.
11. Polis C, Bradley SEK, Bankole A, Onda T, Croftand TN, Singh S. Contraceptive Failure Rates in the Developing World: An Analysis of Demographic and Health Survey Data in 43 Countries New York: Guttmacher Institute, 2016.
12. Vassall A, Sweeney S, Kahn J, Gomez G, Bollinger L, Marseille E, Herzel B, DeCormier Plosky W, Cunnama L, Sinanovic E, Bautista-Arredondo S, Group GTA, Group GS, Harris K, Levin C. Reference Case for Estimating the Costs of Global Health Services and Interventions 2017. Available from: <https://ghcosting.org/pages/standards/reference_case>.

# APPENDIX I COVID-19 RESPONSE PROCEDURES

In response to the COVID-19 pandemic and policy changes issued by the Kenyan Ministry of Health (MOH), we are ensuring study procedures protect the health and safety of study participants and staff. Study visits align with routine patient clinical care schedules, and all participants and staff will follow MOH guidelines within the facility to reduce risks of potential exposure i.e. use of personal protective equipment, appropriate distancing with participants, reducing contact with commonly touched surfaces, handwashing and environmental cleaning. We will provide participant reimbursement via M-PESA to minimize touch points between staff and participants. All data will be collected by study staff on tablets to which only they will have access and will follow disinfection procedures. Participants will not be required to touch any study materials.

**Revisions to Data Collection Procedures**

Any in-person home visits for follow-up or clinic visits for in-depth interviews will be suspended until social distancing measures have been rescinded by the Kenyan government. Follow-up data collection will occur via study visits aligned only with routine clinical care or phone as already noted in this protocol. Verbal autopsies and in-depth interviews will be conducted via phone until in-person study-specific visits are approved by appropriate authorities.
